# Shift work promotes adipogenesis via cortisol-dependent downregulation of EGR3-HDAC6 pathway

**DOI:** 10.1038/s41420-024-01904-9

**Published:** 2024-03-11

**Authors:** Xinxing Wan, Linghao Wang, Md Asaduzzaman Khan, Lin Peng, Keke Zhang, Xiaoying Sun, Xuan Yi, Zhouqi Wang, Ke Chen

**Affiliations:** 1 Department of Endocrinology, The Third Xiangya Hospital of Central South University, Changsha, 410013 Hunan PR China; 2 Department of Biochemistry and Microbiology, North South University, Dhaka, 1229 Bangladesh; 3 Pulmonary Department, Chobanian & Avedisian School of Medicine, Boston University, Boston, MA 02118 USA; 4 Department of Nephrology, The First Hospital of Changsha, Changsha, 410005 Hunan PR China

**Keywords:** Obesity, Predictive markers

## Abstract

The disruption of circadian rhythms caused by long-term shift work can cause metabolic diseases such as obesity. Early growth response 3 (EGR3) is a member of early growth response (EGR) family, which is involved in several cellular responses, had been reported as a circadian rhythm gene in suprachiasmatic nucleus. In this research, EGR3 was found to be widely expressed in the different tissue of human and mice, and downregulated in adipose tissue of obese subjects and high-fat diet mice. Moreover, EGR3 was found negatively regulated by cortisol. In addition, EGR3 is a key negative modulator of hADSCs and 3T3-L1 adipogenesis via regulating HDAC6, which is a downstream target gene of EGR3 and a negative regulator of adipogenesis and lipogenesis. These findings may explain how circadian rhythm disorder induced by shift works can cause obesity. Our study revealed a potential therapeutic target to alleviate metabolic disorders in shift workers and may provide better health guidance to shift workers.

## Introduction

Circadian rhythm can maintain orderly and stable life activities by regulating the glucose and lipid metabolism, hormone secretion and other physiological and metabolic pathways [1–3]. Disruption of circadian rhythm such as shift work may increase the risk of obesity, diabetes, metabolic syndrome, cardiovascular disease, and thus affect human health [4, 5].

A stable circadian rhythm is extremely important for human health, but due to the development of society and division of labor, shift work has gradually developed into a common working hour system, which is very common in medical, transportation, manufacturing and service sectors. Disrupting of circadian rhythm due to shift work, complicating metabolic syndrome and tumors have been reported frequently and have attracted extensive attention from researchers [6–10]. Suprachiasmatic nucleus (SCN) acts as a pacemaker for central circadian rhythm regulation, and it is necessary for the circadian rhythm of adrenocorticotropic hormone (ACTH) and glucocorticoid (GC) release [11].

The peripheral circadian rhythm biomarker for adipogenesis was not clearly, variety of circadian rhythm genes, such as Bmal1 and PER3 were reported can directly regulate adipogenesis [12, 13]. Early growth response (EGR) family and regulated by melatonin and are involved in several cellular responses, which are known to be related to circadian rhythm [14–17]. EGR1 is one of the early downstream nuclear targets for changes in the extracellular stimulation [18, 19], which could be rapidly induced by cytokines, hormones, and stress signals [20]. EGR1 could suppress the transcription of adipose triglyceride lipase and adipogenesis [21, 22]. The expression of uncoupling protein 1 (Ucp1) was restrained in the subcutaneous fat of EGR1 mutant mice, and EGR1 could alter beige adipocyte and promote white adipocyte differentiation in the stem cells of mouse [23]. EGR2 is proved to be an early regulators of the white adipogenic program [24], which can stimulate adipogenesis through C/EBPβ-dependent or -independent mechanisms [25]. EGR3 also has a zinc finger-type transcription factors, however, its behavior in the fat metabolism or adipogenesis is still not clear [26–28]. Here we proved the expression of EGR3 is decreased in visceral adipose tissues (VATs) of night shift nurses, and the inhibitor of EGR3 of adipogenesis could be reversed by histone deacetylase 6 (HDAC6).

HDAC6 is one of the subtypes of HDAC family, which is preponderantly localized in the cytoplasm [29, 30]. HDACs has E3 ligase activity and deacetylase activity; beside these functions, HDAC6 has a special C-terminal ubiquitin-binding domain, which can directly interact the proteins should be degraded by proteasome [31]. Nowadays, HDAC6 has been proved to play an important role in the formation of lipid droplet and lipid storage in adipocytes. This indicates that HDAC6 could affect the metabolism of whole body and substantiated the HDAC6 is important regulator of adipose tissue [32]. Mice with specific depletion of *hdac6* in adipose showed elevated lipid storage and insulin resistant [33, 34]. EGR3 has been reported to bind to the promoter of HDAC6 to promote its expression [35].

In this research, we attempted to find the relationship between EGR3 abnormalities caused by circadian disorders in night shift nurses and related obesity, and tried to provide a suitable solution to alleviate the metabolic abnormalities caused by circadian disorders.

## Results

### The body weight gain and metabolic dysfunction in the night shift nurses

To study the relationship between shift work and metabolic parameter, 520 nurse volunteers were recruited. We collected basic information such as frequency of shift work and years of shift work through questionnaires and divided into day shift nurse group and night shift nurse group. The body weight and BMI were significantly increased in night shift nurses compared to day shift nurses; however, average sleep time in a week was found shorter than day shift nurses; the metabolic parameter revealed that cholesterol, 8am ACTH and cortisol levels were increased in night shift nurses, but triglycerides, HDL, FBG, FINS, and 8am melatonin were found statistically indifferent (Table 1). Our results implied that shift work-induced circadian disorder related to metabolic disturbances.

**Table 1:** The clinical data of day shift nurses and night shift nurses.

Clinical data	Day shift nurses (*n* = 257)	Night shift nurses (*n* = 263)
Age (year)	40.6 ± 5.2	38.3 ± 4.6
Height (cm)	160.4 ± 5.7	159.3 ± 5.1
Body weight (kg)	53.6 ± 6.7	58.5 ± 6.3*
BMI (kg m^−2^)	20.9 ± 2.8	23.2 ± 2.3*
Working years (year)	18.6 ± 3.1	17.4 ± 3.3
Night shift years (year)	0	16.5 ± 3.5
Night shift frequency (time/week)	0	1–2
Sleep time (h/week)	56 ± 3.1	50 ± 3.9*
Smoke	No	No
Excessive drinking	No	No
Triglyceride (mmol/L)	1.23 ± 0.58	1.39 + 0.37
Cholesterol (mmol/L)	4.11 ± 1.64	6.65 ± 1.37*
HDL (mmol/L)	1.43 ± 0.21	1.33 ± 0.15
FBG (mmol/L)	5.2 ± 0.21	5.7 ± 0.32
FINS (μU/ml)	5.3 ± 1.7	5.6 ± 1.2
8am ACTH (pg/ml)	15.37 ± 3.56	19.88 ± 3.25*
8am cortisol (μg/dL)	14.83 ± 4.49	19.21 ± 5.21*
8am melatonin (pg/ml)	13.83 ± 7.38	11.21 ± 6.17

*p *<* *0.05* vs day shift nurses.

### EGR3 was significantly decreased in the VATs of night shift nurses

**Fig. 1:**
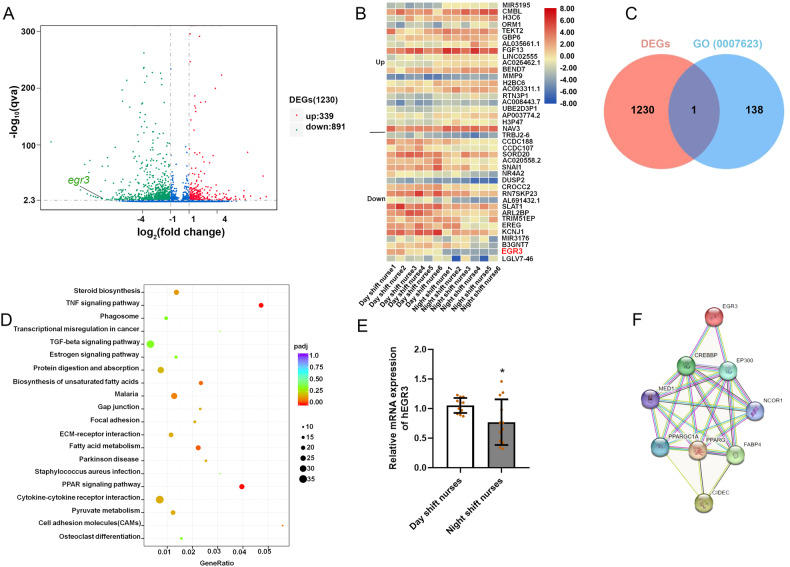
The expression of EGR3 was decreased in the VATs of night shift nurses. **A** The volcano plot of RNA-seq. **B** Heatmap of top 40 representing the DEGs of VATs in day shift nurses and night shift nurses. Red and blue respectively represent upregulation and downregulation expression, *n* = 6. **C** Intersection with our RNA-seq with circadian rhythm genes in public GO database (GO007623). **D** The pathway analysis of RNA-seq. **E** The PCR results of EGR3 in the VATs of day shift nurses (*n* = 16) and night shift nurses (*n* = 12). **F** The relationship predicted by STING. *p < 0.05 vs day shift nurse group.

To further investigate the differentially expressed genes (DEGs) in VATs between 6 day shift nurses and 6 night shift nurses, RNA-seq was used. A total 1230 DEGs were identified, of which 339 were upregulated and 891 were downregulated in night shift nurses (Fig. 1A). The heatmap showed the most upregulated 20 genes and the most downregulated 20 genes (Fig. 1B). Through we did not find the change of circadian rhythm pathway in GO analysis, we searched the data base of circadian rhythm genes in public GO (GO:0007623). (http://amigo.geneontology.org/amigo/term/GO:0007623), and intersection with our RNA-seq results, only the expression of EGR3 was found in DEGs of VATs (Fig. 1C). What more, the heatmap showed EGR3 is one of the most downregulated genes (FDR = 9.57E-6) in the results of RNA-seq (Fig. 1B). The pathway analyzed found lipid metabolic and adipogenesis pathways such as lipid synthesis, steroid synthesis, unsaturated acid synthesis and PPAR pathway were enriched, indicating that shift work is a key factor in the development of obesity (Fig. 1D). Next, the expression of EGR3 was further verified that it is downregulated in VATs of 12 night shift nurses compared to 16 day shift nurses (Fig. 1E). Lastly, the STING predicted the EGR3 was closely related to PPARγ, FABP4 and cell death-inducing DFFA-like effector c (CIDEC) (Fig. 1F). We supposed that EGR3 may be the biomarker in the process of obesity caused by rhythm disturbances.

### The EGR3 was decreased in the SATs and VATs of obese subjects

**Fig. 2:**
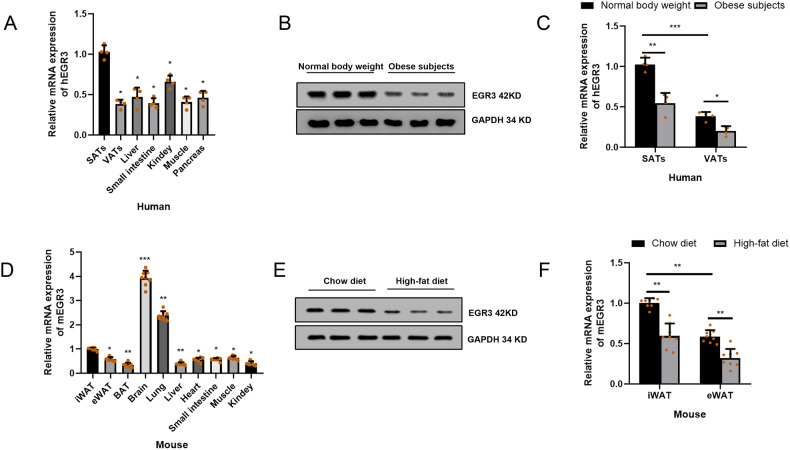
The expression of EGR3 decreased in obese subjects. The expression of EGR3 mRNA was analyzed in different tissues of human (**A**) and EGR3 protein was downregulated in VATs of human (**B**). **C** The expression of EGR3 mRNA was lower in SATs and VATs of obese people (BMI ≥ 28.0 kg·m^2^) than that in normal weight subjects (BMI 18.5–23.9 kg m^2^). **D** The expression of EGR3 mRNA was analyzed in different tissues of mice. **E** The expression of EGR3 protein of iWAT and mRNA (**F**) of iWAT and eWAT was lower in high-fat diet mice. *p < 0.05, **p < 0.01, ***p < 0.001 vs SATs or iWAT.

Although it had been proved that EGR3 as a circadian rhythm gene in central SCN induced by light [36], whether the EGR3 is expressed in adipose tissue is not clear. We found that EGR3 was commonly expressed in the human subcutaneous adipose tissues (SATs), VATs, liver, kidney, muscle small intestine and pancreas (Fig. 2A). Interestingly we found that the protein level of EGR3 expression in the VATs and mRNA expression in SATs and VATs was reduced of obese subjects (Fig. 2B, C). Similarly, we found that EGR3 was highly expressed in mouse brain tissue and commonly expressed in mouse lung, liver, heart, small intestine, kidney, muscle, iWAT, eWAT and brown adipose tissue (BAT) (Fig. 2D). The EGR3 protein expression in iWAT and mRNA expression of iWAT and eWAT was found downregulated in high-fat fed mice (Fig. 2E, F). Our results support that EGR3 may has a physiological role in the function of the peripheral circadian rhythm system and EGR3 may be a potential suppressor of obesity.

### EGR3 is a key suppressor of adipogenesis in vitro

**Fig. 3:**
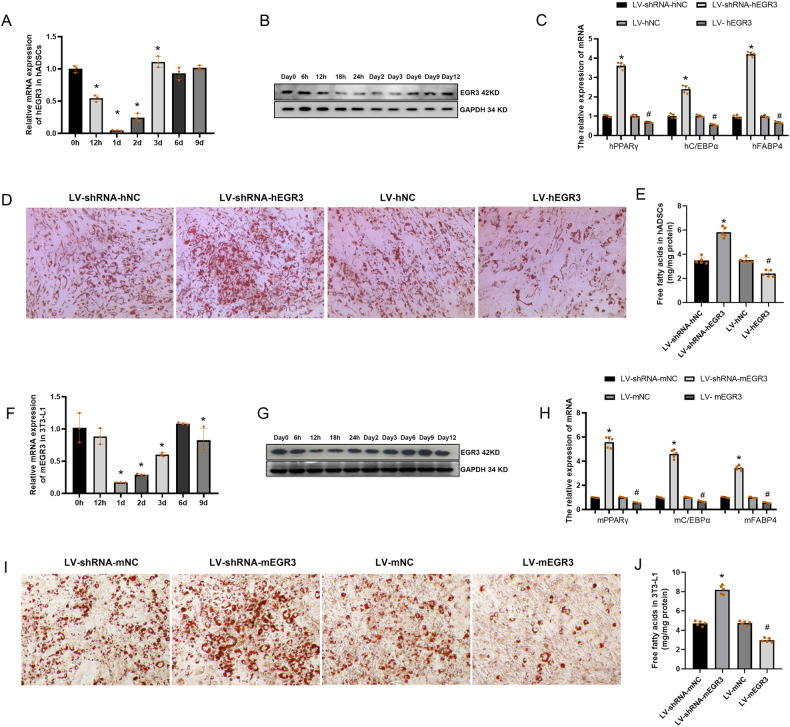
The EGR3 negatively regulates adipogenesis in hADSCs and 3T3-L1. **A** PCR and Western blot (**B**) confirmed that EGR3 was downregulated in the early stage of hADSCs adipogenesis. **C** Overexpression and silencing of EGR3 affected the expression of related genes for hADSCs adipogenesis on day 6 (PPARγ/CEBPα/FABP4). **D** Overexpression and silencing of EGR3 affected the early differentiation of hADSCs (Day 6, Oil Red O) and the content of FFA (**E**). **F** PCR and Western blot (**G**) confirmed that EGR3 was downregulated in the early stage of 3T3-L1 adipogenesis. **H** Overexpression and silencing of EGR3 affected the expression of related genes for 3T3-L1 adipogenesis on day 6 (PPARγ/CEBPα/FABP4). **I** Overexpression and silencing of EGR3 affected the early adipogenesis of 3T3-L1 (Day 6, Oil Red O) and the content of FFA (**J**). *p < 0.05 vs LV-shRNA-h/mNC, ^#^*p* < 0.05 vs LV- h/mNC.

To explore whether EGR3 can regulate adipogenesis, firstly, the EGR3 mRNA and protein expression were tested during the hADSCs adipogenesis, the results showed that EGR3 was decreased in the early stage of adipogenesis and there was a gradual recovery in middle and later stages of adipogenesis (Fig. 3A, B). Moreover, Oil Red O staining and FFA content in media results indicated that silencing of EGR3 increased hADSCs adipogenesis, however, overexpression of EGR3 decreased hADSCs adipogenesis (Fig. 3C, D). Meanwhile, silencing or overexpression of EGR3 resulted in increasing or decreased markers of adipogenesis, such as, PPARγ, C/EBPα and FABP4, respectively, further demonstrating the adipogenic inhibitory capacity of EGR3 during adipogenesis (Fig. 3D, E). Similar results were verified in 3T3-L1 adipogenesis, where the mRNA and protein expression were only downregulated in early stage of adipogenesis (Fig. 3F, G), and downregulation or upregulation of EGR3 enhanced or inhibited PPARγ, C/EBPα and FABP4 expression and Oil Red O staining in 3T3-L1 adipogenesis (Fig. 3H, I), and elevated FFA or attenuated content, respectively (Fig. 3J).

### The rhythm of EGR3 is negatively related to cortisol

**Fig. 4:**
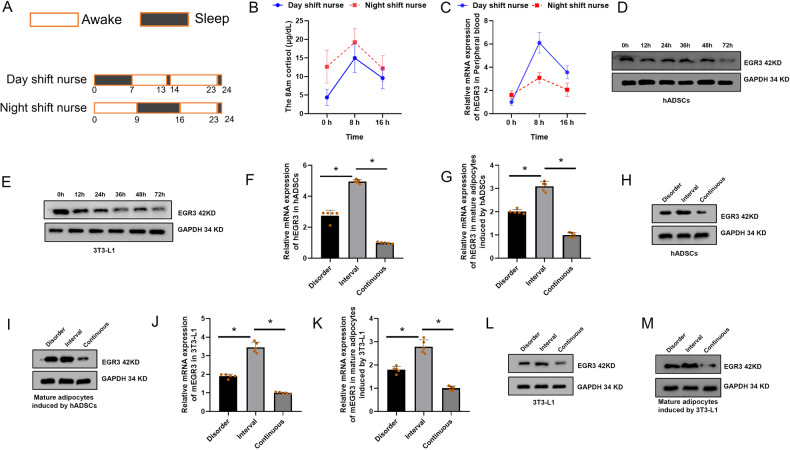
The sleep pattern differences between day shift nurses and night shift nurses. **A** Sleep patterns and diagrams of day shift nurses and night shift nurses. **B** The circadian rhythm of cortisol in the nurses. **C** The expression of EGR3 in the peripheral blood of day shift nurse and night shift nurse. **D** Continuous dexamethasone intervention downregulates EGR3 in hADSCs. **E** Continuous dexamethasone intervention downregulates EGR3 in 3T3-L1. **F** PCR confirms that dexamethasone continuously downregulated EGR3 expression in continuous group and a 4-h interval (disorder group) simulating a night-shift compare to 12-h interval (interval group) simulating a day-shift in hADSCs and mature adipocytes induced by hADSCs (**G**). **H** Western blot confirm that dexamethasone is continuously downregulated EGR3 expression, in a 4-h interval simulating a night-shift in hADSCs and mature adipocytes induced by hADSCs (**I**). **J** PCR confirmed that dexamethasone was continuously downregulated EGR3 expression, in a 4-h interval simulating a night-shift in 3T3-L1 and mature adipocytes induced by 3T3-L1 (**K**). **L** Western blot confirmed that dexamethasone continuously downregulated EGR3 expression, in a 4-h interval simulating a night-shift in in 3T3-L1 and mature adipocytes induced by 3T3-L1 (**M**). *p < 0.05.

The sleep patterns of day shift nurses and night shift nurses showed obvious difference (Fig. 4A). As a key hormone of the central rhythmic system, cortisol regulates the peripheral rhythmic system, and cortisol secretion is highly rhythmic. Our results revealed that cortisol secretion rhythmicity was not affected in night shift nurses, but cortisol levels were commonly found higher in night shift nurses than day shift nurses at three time points, even cortisol levels still cannot be descended to the lowest levels at 4 pm after night shift nurses sleep for 7 hours at 9 am to 4 pm (Fig. 4B). In addition, we further analyzed the EGR3 mRNA expression in peripheral blood of night shift nurses and day shift nurses. Our results demonstrated that EGR3 expression is downregulated in night shift nurses compared to day shift nurses in three-time points (Fig. 4C).

To further investigate the potential regulation relationship between EGR3 and cortisol in vitro, hADSCs and 3T3-L1 were cultured in 1 μM dexamethasone for 0 h to 72 h, respectively. The EGR3 expression was gradually downregulated during adipogenesis, implying that cortisol negatively regulates EGR3 in vitro (Fig. 4D, E). To explore whether shift work mediated-cortisol dysfunction induced EGR3 down-expression, the hADSCs and 3T3-L1 were culture in 1 μM dexamethasone for 48 h every 12 h interval (interval group) to mimic the normal rhythm, interval dexamethasone cultured for 48 h in every 4 h interval (disorder group) to mimic night shift nurses, and persistent dexamethasone (continuous group) was added to mimic Cushing’s syndrome. Our results showed that the hADSCs treated with 4 h interval dexamethasone could inhibit the mRNA and protein expression of EGR3 and continuous dexamethasone-treated cells more significantly inhibited EGR3 expression than 12 h interval dexamethasone-treated hADSCs and mature adipocytes induced by hADSCs (Fig. 4F–I). Furthermore, in 3T3-L1 and mature adipocytes induced by 3T3-L1, the results also displayed disorder dexamethasone drastically downregulated EGR3 expression compared to interval 12 h dexamethasone intervene (Fig. 4J–M). Our results may explain that adipogenesis and obesity were caused by disorder rhythm secretion of cortisol via negatively regulated EGR3.

### EGR3 negatively regulated adipogenesis dependent on HDAC6

**Fig. 5:**
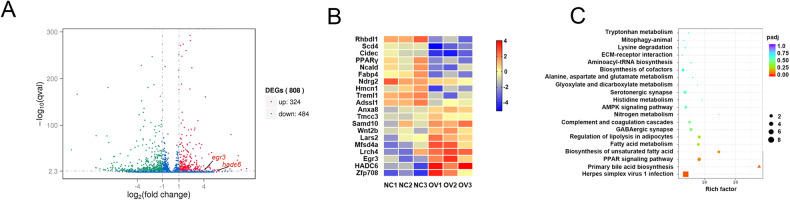
The RNA-seq results of over-expressing EGR3. **A** The volcano plot of RNA-seq. **B** Heatmap showed the 20 related genes with significant differences. Red and blue respectively represent upregulation and downregulation expression, *n* = 3. **C** The pathway analysis of RNA-seq.

**Fig. 6:**
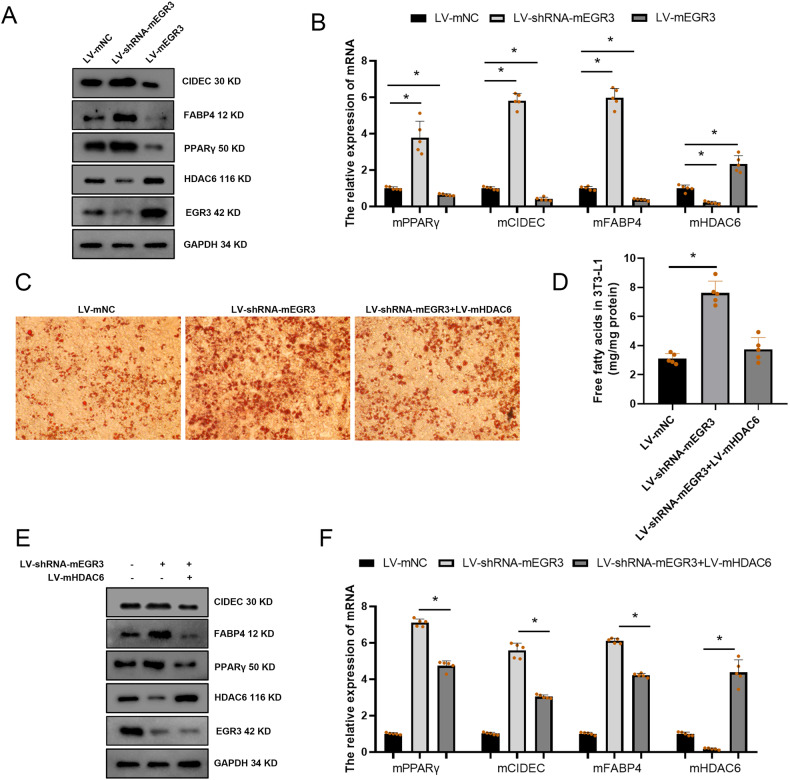
EGR3 effect on adipogenesis. **A** Western blot and PCR (**B**) assays verified the expression of PPARγ, CIDEC, FABP4 and HDAC6 after overexpression or silencing of EGR3 in 3T3-L1. **C** The rescue experiment using Oil Red O staining assay and FFA content (**D**) were used to detect the differentiation phenotype after 3T3-L1 was induced for 6 days. **E** The rescue experiment using western blot and PCR (**F**) assay verified the changes of lipogenesis and adipogenesis molecules (PPARγ, CIDEC, FABP4) after overexpression of HDAC6 and silencing of EGR3. *p < 0.05.

**Fig. 7:**
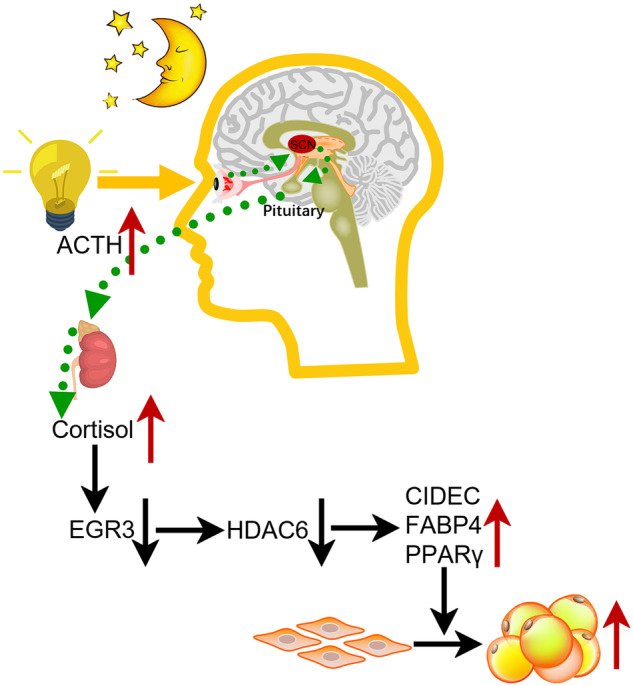
The schematic diagram summarizes the mechanism of shift work causing cortisol disturbance and downregulation of EGR3 to induce adipogenesis via HDAC6.

To assess the mechanism of how EGR3 regulates adipogenesis, RNA-seq was used to explore. The DEGs in 3T3-L1 were transfected using EGR3 (FDR = 7.26E-6) overexpression plasmid. There were 324 upregulated and 484 downregulated DEGs in overexpressed EGR3 compared to NC group (Fig. 5A), among them, the heatmap also showed the FABP4 (FDR = 9.73E-14) and PPARγ (FDR = 1.21E-6) were decreased while HDAC6 (FDR = 5.1E-4) was increased in the 3T3-L1, when transfected with LV-EGR3 (Fig. 5B). The pathway analysis showed that DEGs were enriched in fatty acid metabolism and PPAR pathway (Fig. 5C). The western blot results also proved that FABP4, PPARγ and CIDEC were decreased and HDAC6 was increased when EGR3 was overexpressed and increased when EGR3 was silenced (Fig. 6A, B). HDAC6, a critical negative regulatory factor of adipogenesis and lipogenesis, was reported as a target gene of EGR3 through targeting its promoter [32]. In rescue experiments, HDAC6 can partially reverse the adipogenesis and lipogenesis induced by silencing of EGR3 in Oil Red O staining and FFA concentration (Fig. 6C, D). Meanwhile, the expression of FABP4, PPARγ and CIDEC could also be partially reversed in the co-transfected LV-shRNA-mEGR3 and LV-HDAC6 overexpression (Fig. 6E, F). These results revealed the inhibitory effect of EGR3 on adipogenesis and lipogenesis dependent on HDAC6. A proposed biological model showing how the shift work mediated cortisol secretion dysfunction and induced EGR3-HDAC6 downregulation promoting adipogenesis is summarized in Fig. 7.

## Discussion

Shift work is a production system that ensures the stable operation of the medical, industrial, transport and many other sectors in modern society. However, the disruption of circadian rhythms caused by shift work should be noticed. To avoid diseases caused by circadian rhythm disorders, the clock gene is receiving more attention from researchers [37, 38]. EGR3, a zinc finger transcription factor, has been proved to be induced by light restricted to the ventral SCN [36, 39]. As the member of EGR family, EGR1 and EGR2 are known to be the critical regulator of peripheral circadian rhythm in human and other animals [40, 41]. Although the rhythm of EGR3 in peripheral tissues had few reports, we speculated that EGR3 might be closely related to peripheral circadian rhythms. In our study, we found EGR3 is universally expressed in most of organisms and for the first time proved that the expression of EGR3 in the VATs is only rhythmically variable gene by transcriptome sequence, indicating that the EGR3 cloud function as peripheral clock gene in peripheral tissues for the universal expression. This implies that the EGR3 could be the critical circadian rhythms regulator for its role in modifying clock gene expression in VATs.

As a critical hormone of neuroendocrine system, cortisol secretion is highly rhythmic, which is highest in the morning and lowest late at night. The circadian rhythm cortisol release was regulated by SCN-hypothalamo-pituitary-adrenal axis (HPA) [42]. Cortisol can promote obesity by influencing leptin sensitivity and insulin resistance, and the abnormally high cortisol in plasma cause abnormal obesity and Cushing’s syndrome [43].

As the human glucocorticoid receptor isoform alpha (GR) contains 2 structural domains of zinc finger, we presumed that the expression of EGR3 could closely related to SCN-ACTH controlled cortisol. Leclerc et al. reported that cortisol inhibits EGR3 in osteoblast [44–46]. We primarily explored whether cortisol can affect the expression and rhythms of EGR3 by detecting the plasma in 520 nurses and proved the night shift nurses secreted more cortisol than the day shift nurses, while the expression of EGR3 in the night shift nurses decreased, which help to predicting obesity risk as biomarker. This proved that the cortisol in plasma was negatively related with the expression of EGR3 in peripheral blood.

As some clock genes such as Bmal1 and PER3 can directly regulate lipid adipogenesis [47–50], there is evidence that the expression of EGR3 is reduced in high-fat fed mice, implying that the EGR3 is negatively related with adipogenesis [51]. EGR3 is the only rhythmically variable gene in VATs between the shift nurses, and so, we conclude that EGR3 may be responsible for causing obesity induced by shift work. To confirm, we transfected EGR3 shRNA or overexpression plasmid to hADSCs and 3T3-L1, and found that EGR3 could negatively regulate adipogenesis, which partially explained cortisol can cause abnormal obesity. Then we further proved hADSCs and 3T3-L1 with 12 hours of alternating dexamethasone treatment expressed most EGR3 as simulating normal rhythm group, while the cells treated with continuous and disorder dexamethasone group presented the least EGR3. These results explain that normal people with rhythm cortisol have low risk of obesity, while continuous and disorder-high cortisol in plasma causes abnormal obesity and decreased EGR3.

EGR3 can negatively regulate adipogenesis through fatty acid metabolism and PPAR pathway, which we confirmed by RNA-Seq, and this function was found cortisol-independent, however, the mechanism of EGR3 regulation of adipogenesis is not clear. To clarify the possibility of the mechanism, bioinformatics tools were used to predict possible pathways and targets for EGR3. Some key transcription factors in adipogenesis and fat metabolism such as PPARγ and FABP4 are closely related to EGR3, and CIDEC, and a recently reported target of HDAC6 was also found to be regulated by EGR3 [32]. As HDAC6 is predominantly localized to the cytoplasm and loss of HDAC6 leads to significant age-dependent ectopic fat accumulation, and there are reports that CIDEC is a specific downstream target of HDAC6 [32], CIDEC contains a PPRE in the region of promoter and can be directly active by PPARγ, which played an important role in the regulation of lipid accumulation in adipocytes [52]. Expression of CIDEC is the most efficacious indicator of strong PPARγ activation [53–55]. HDAC6 could abolish the acetylation of CIDEC at K56 through PCAF, which could decrease the stability of CIDEC and elevate lipid droplet fusion and growth [32]. In our study, after overexpression of EGR3 by LV-mEGR3 in 3T3-L1 cells, we found the expression of EGR3 was positively related to HDAC6, and improving the expression of HDAC6 could alleviate the adipogenesis of LV-shRNA-EGR3. These results prove that the adipogenesis inhibitor of EGR3 is positively dependent on HDAC6, and the restorage of HDAC6 could reverse the inhibitory effect of EGR3 on lipogenesis and adipogenesis, which explains the mechanism of EGR3 inhibition of adipogenesis.

In the present study, firstly we identified that EGR3 can function as a cortisol-dependent peripheral clock gene, and we proved that EGR3 can inhibit adipogenesis in vitro, which can explain the continuous high cortisol in plasma causing abnormal obesity. This can also prove that EGR3 can work as an important regulator of rhythm disturbance leading to the development of obesity, which can inhibit adipogenesis through HDAC6. In general, we conclude EGR3 is a peripheral circadian rhythm biomarker for adipogenesis, which can help relieve metabolic disorders caused by shift work, and EGR3 can be a potential target for the abnormal obesity caused by continuous high cortisol in plasma.

## Materials and methods

### Volunteer recruitment, and collection of data and tissue samples

520 female nurse volunteers (263 day shift nurses and 257 night shift nurses) from the Third Xiangya Hospital of Central South University and The First Hospital of Changsha were recruited. The basic information was obtained by questionnaires; the physical data such as height, body weight was obtained by anthropometric measurement and BMI was calculated according to kilograms divided by the square of height in meters. The VATs in the omentum majus from day shift nurses (*n* = 16) and night shift nurses (*n* = 12) were collected; cholesterol, triglyceride and high-density lipoprotein (HDL) were measured by Department of Clinical Laboratory Center, and fasting blood glucose (FBG), fasting insulin (FINS), ACTH, cortisol and melatonin were tested by Department of Endocrine Experiment Center of The Third Xiangya Hospital of Central South University.

In addition, human tissue samples such as abdominal subcutaneous adipose tissues (SATs), VATs, liver, small intestine, kidney, muscle, and pancreas were collected from 4 unrelated patients who suffered severe trauma. Partial SATs and VATs were collected from four normal weight individuals (BMI:18.5–23.9 kg m^2^) and 4 obese (BMI ≥ 28 kg m^2^) patients. All human tissue samples were collected from the Department of General Surgery, The Third Xiangya Hospital of Central South University.

All the tissue samples were immediately preserved after surgical collection at −80 °C until analysis. Our study was approved by the Ethics Committee of The Third Xiangya Hospital of Central South University, Changsha, China, (Approval No: 2021-S212), and The First Hospital of Changsha, Changsha, China, (Approval No: 2021-79), and was performed according to the guidelines of Declaration of Helsinki. All participants provided a written informed consent form.

### High-fat diet and sample collection in mice

The animal experiments were performed according to the guidelines of the Ethical Committee for Animal Experiments of Central South University. Briefly, C57BL/6 male mice (4 weeks, *n* = 12) were purchased and fed on a chow diet for suiting the environments. After 2 weeks, 6 mice were fed with high-fat diet (60% fat, 20% carbohydrates, and 20% proteins) (D12492, Research Diets, New Brunswick, NJ), and another 6 mice were continued with chow diet for 12 weeks. The tissue samples including the inguinal white adipose tissue (iWAT), epididymal white adipose tissue (eWAT), liver, pancreas, etc. were collected and preserved at −80 °C to explore the mRNA expression of mEGR3. All mice samples were collected at 8 h of circadian time.

### The isolation and differentiation of hADSCs

The isolation and differentiation of human adipose tissue-derived stromal cells (hADSCs) were conducted as described in our previously published study [12]. Briefly, 10 g of fresh abdominal VATs were washed in and cut into 1 mm × 1 mm size, then incubated in collagenase I solution (Gibco, Life Technology, China) for 1.5 h at 37 °C. The treated sample was cultured in DMEM/F12 (Life Technologies, Carlsbad, CA, USA) and supplemented with 10% fetal bovine serum (FBS; Life Technologies). For hADSCs differentiation, confluent hADSCs were cultured in inducing medium (DMEM/F12 supplemented with 1 μM dexamethasone, 10 μM insulin, 0.5 mM isobutylmethylxanthine (IBMX), and 200 μM indomethacin (all provided by Sigma, St. Louis, MO, USA). The medium was replaced every 2 days.

### 3T3-L1 cell culture and differentiation

3T3-L1 cells were bought from Wellbiology (Changsha, China) and cultured with high glucose DMEM (Life Technologies, Carlsbad, CA, USA) containing 10% FBS and 100 U/ml penicillin and 100 μg/ml streptomycin. When cells reached confluence, the cells were induced by DMEM containing 1 μM dexamethasone, 10 μM insulin and 0.5 mM IBMX. After 48 h, the medium was changed to DMEM containing 10% FBS and 10 μM insulin for every 48 h.

### Oil Red O staining and free fatty acid (FFA) measure

The cells were fixed by 4% paraformaldehyde and washed by PBS three times, the Oil Red O (Sigma, St. Louis, MO, USA) was added to the plates and incubated at room temperature for 10 min and washed by water three times. The images were captured by microscope (Leica DM IL LED Fluo, Germany). FFA in an extracellular medium were tested by fluorescent quantification kit (MAK044, Sigma).

### Cell transfection

The mouse and human lentiviral EGR3-overexpressing, shRNA, negative control plasmids (LV-shRNA-mEGR3, LV-mEGR3, LV-shRNA-mNC, LV-mNC, LV-shRNA-hEGR3, LV-hEGR3, LV-shRNA-hNC, LV-hNC) and HDAC6-overexpressing plasmid were constructed by Genechem, Shanghai, China. 3T3-L1 or hADSCs were transfected using lentiviral plasmids according to manual when cells were 70%-80% confluent.

### RNA-Seq and data analysis

The RNA-Seq analysis was conducted by Tsingke Biotechnology Co. Ltd (Beijing). To extract the RNA of VATs and 3T3-L1, we utilized the TRizol reagent from Life Technologies (Carlsbad, CA, USA). The data were generated on the Illumina Hiseq 2000/2500 platform and subsequently analyzed using an R package [12]. The raw image data files, obtained from high-throughput sequencing, were processed using CASAVA/Basecall_T7_GPU_1.2.0.26_Centos for Base-Calling and converted into raw sequences. To build a reference genome index, we employed Hisat2 (2.2.1) and used it to align paired-end Clean Reads to the reference genome. The differential expression analysis between sample groups was carried out using DEseq2 (1.26.0) package, which allowed us to identify genes that were differentially expressed between the two biological conditions. Additionally, we performed differential gene enrichment analysis using the clusterProfiler (3.14.3) package. To minimize false positives during the detection of differentially expressed genes, we applied the Benjamini & Hochberg method to adjust the P values (FDR). Finally, we set a threshold of |log2 (foldchange)| > 1 and padj < 0.05 for significant differential expression.

### Real-time RT-PCR

Real-time RT-PCR was performed as described in our previously published study [56]. The total RNA of cells or tissues were isolated and reversed to cDNA. The primer sequences used for qPCR were shown in Supplementary Table 1.

### Western blot assay

Western blotting was performed as described in our previously published study [56]. The total protein of cells was extracted and separated on a 12% sodium dodecyl sulfate-polyacrylamide gel electrophoresis and then transferred to nitrocellulose membranes with a transfer apparatus (Bio-Rad, Hercules, CA, USA). After block, primary antibodies, including EGR3, PPARγ, HDAC6, FABP4, CIDEC, and GAPDH (Proteintech, Wuhan, China) were treated overnight at 4 °C, and second antibody was used for the next ECL (Advansta, Menlo Park, CA, USA) detected.

### Statistical analysis

The clinical data of day shift nurses and night shift nurses were summarized and presented as means ± standard deviation (SD) and analyzed using paired Student’s *t* test. For the data of relative mRNA expression and free fatty acids, Student’s *t* test were carried out for the two groups, and the multiple comparisons were carried out by analysis of variance and were further carried out for post hoc analysis. Data were statistically analyzed by SPSS 17.0 software. The statistically significant level was defined as *p* < 0.05.

### Supplementary information

Original Data File

supplementary table1

## Data Availability

The data are available from the corresponding author on reasonable request.
