# Supplementary material for: Shift work promotes adipogenesis via cortisol-dependent downregulation of EGR3-HDAC6 pathway
Source: Cell Death Discov. 2024 Mar 11;10:129. doi: 10.1038/s41420-024-01904-9 (PMC10928160; doi:10.1038/s41420-024-01904-9)
Supplement: Supplementary file 1 — Original Data File [file 41420_2024_1904_MOESM1_ESM.pdf]

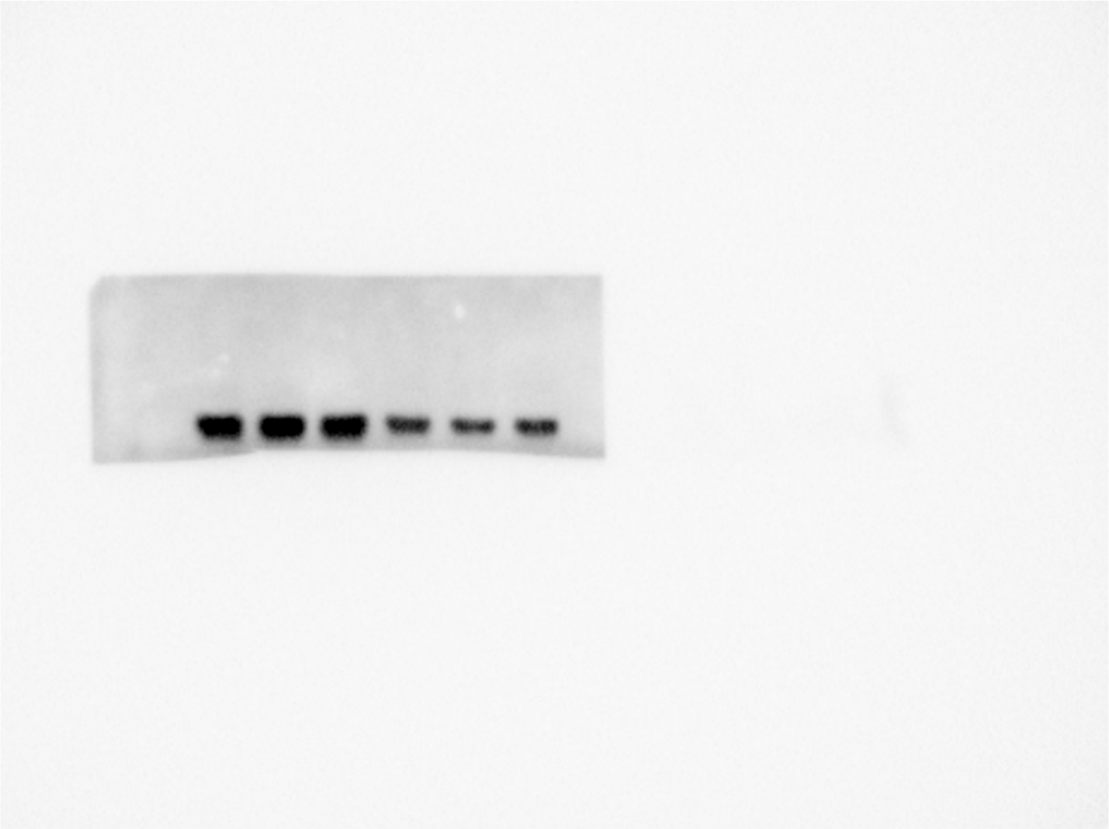

Fig2b egr3

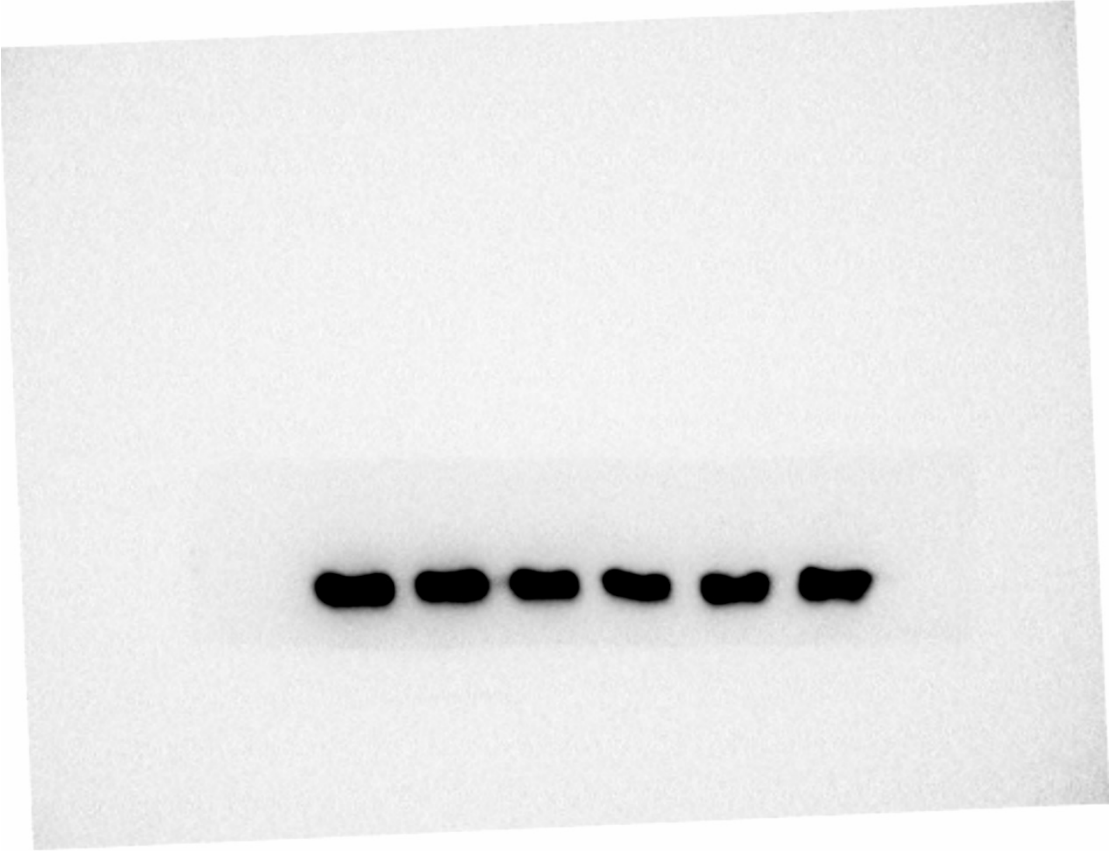

Fig 2b gapdh

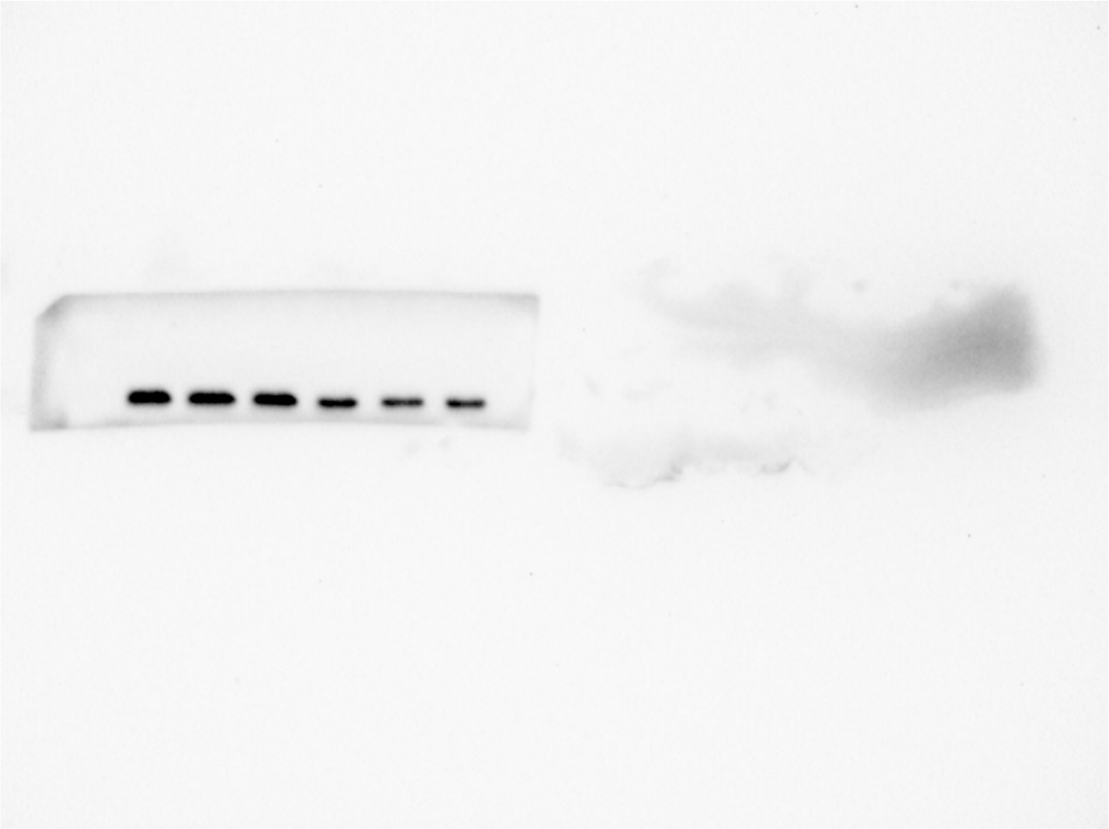

Fig 2e egr3

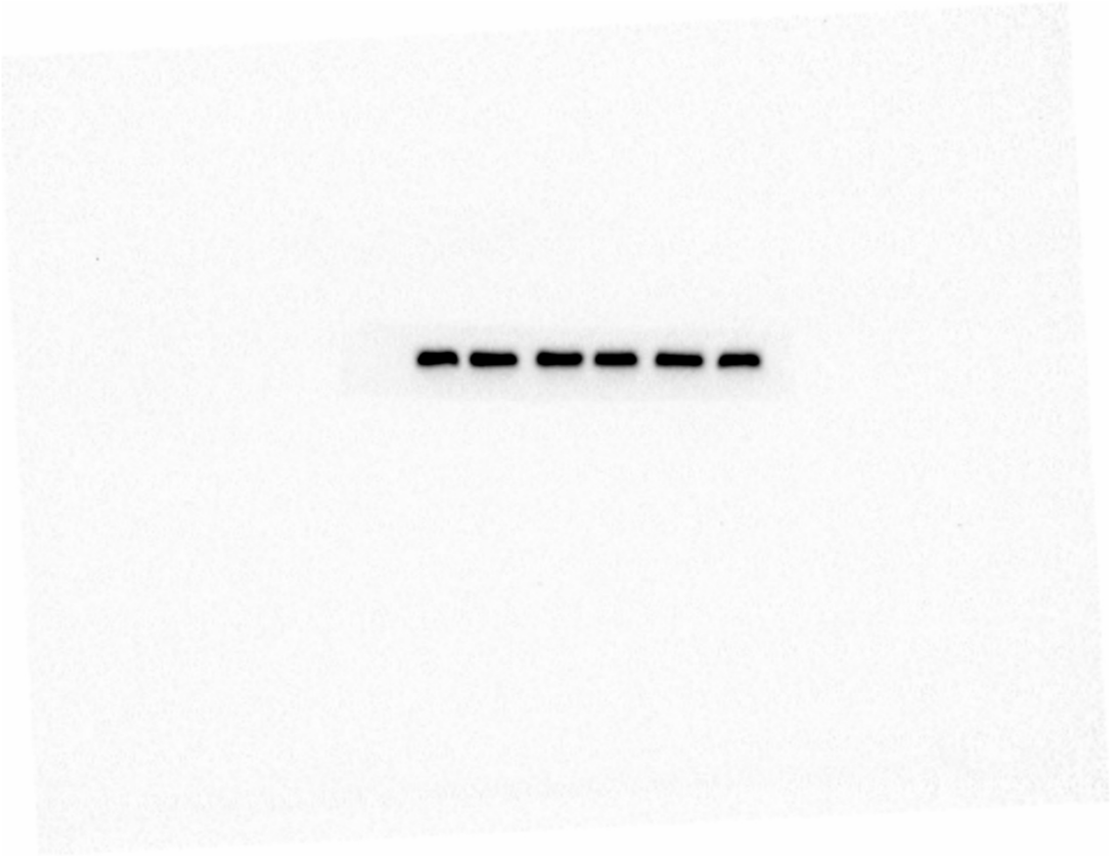

Fig 2e gapdh

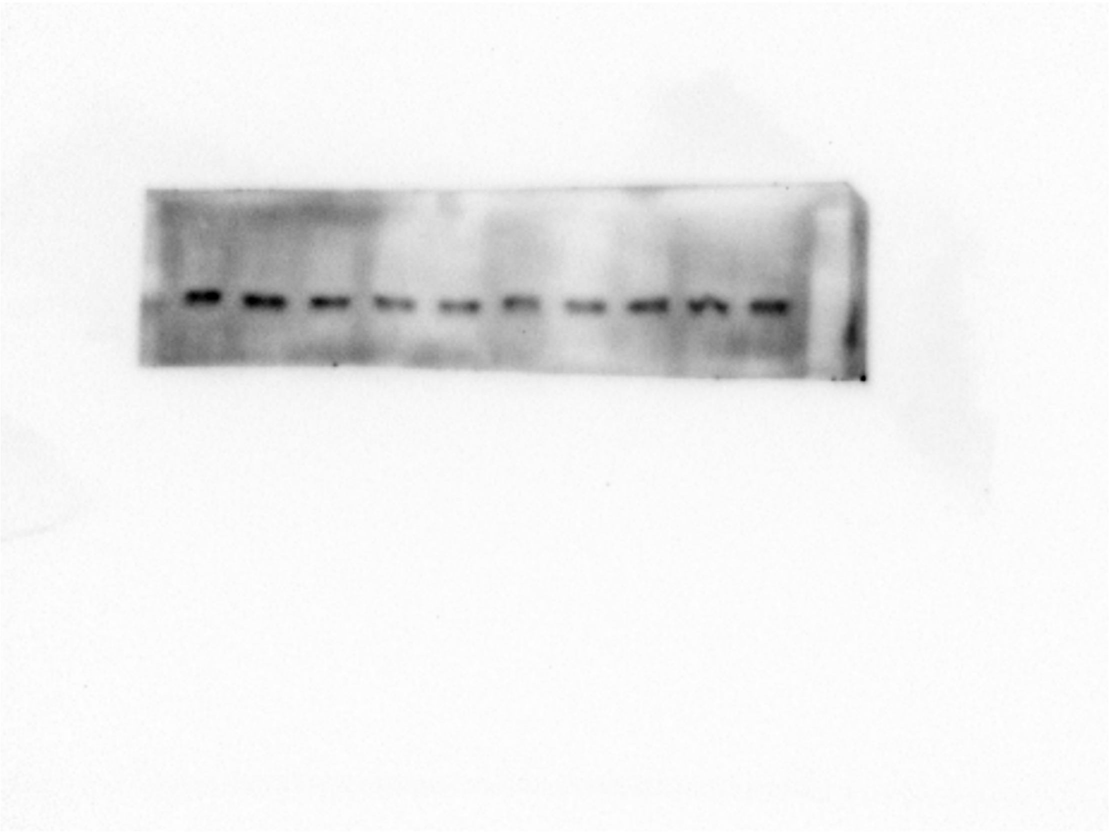

Fig 3b egr3

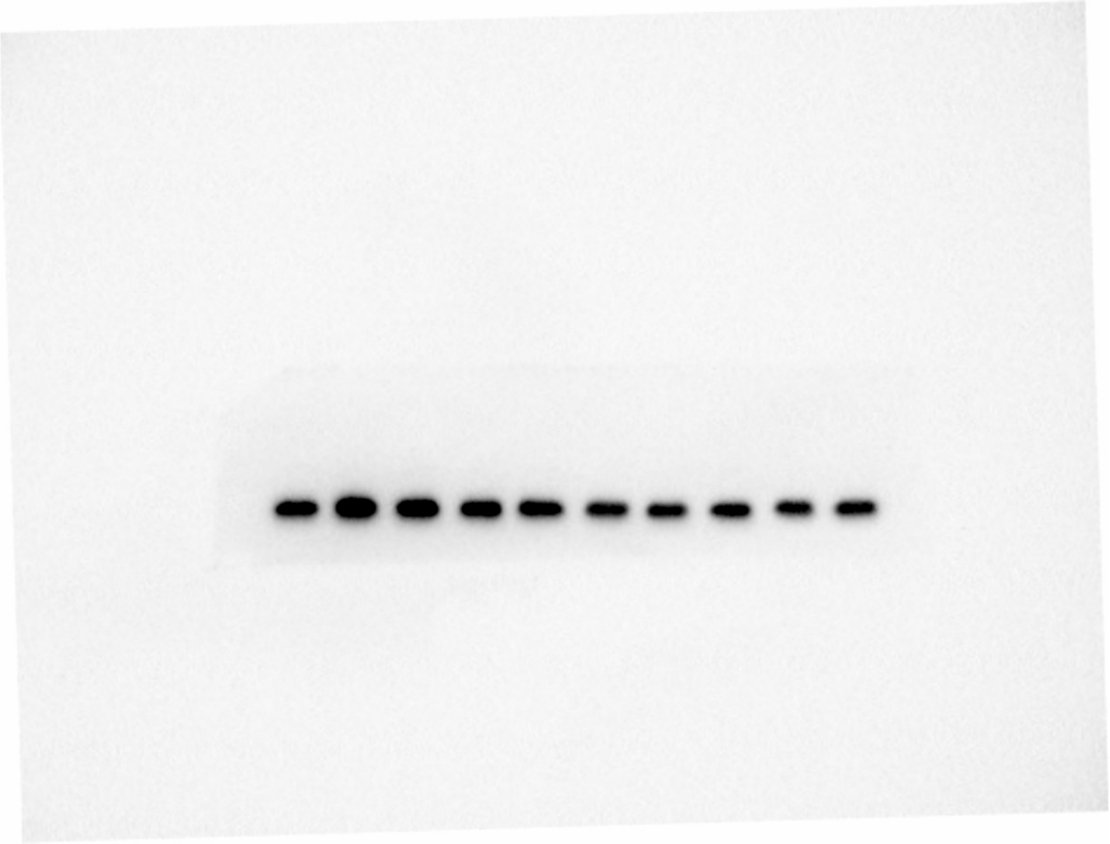

Fig3b gapdh

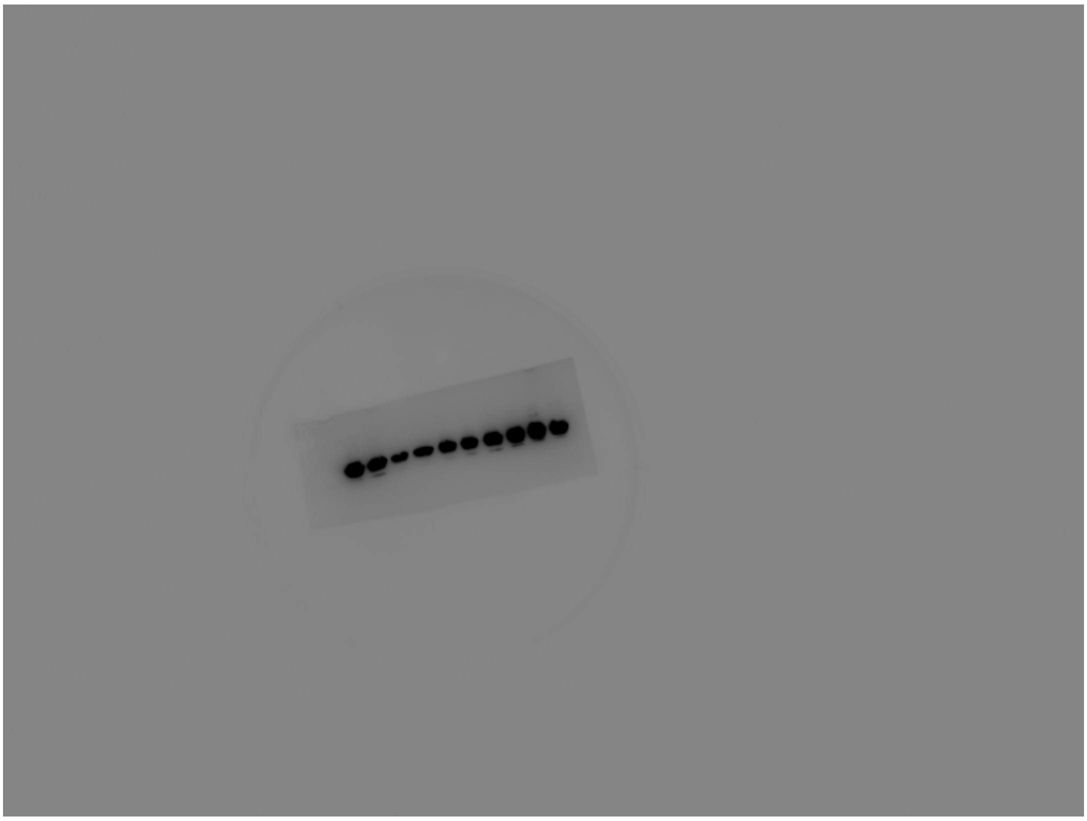

Fig3g egr3

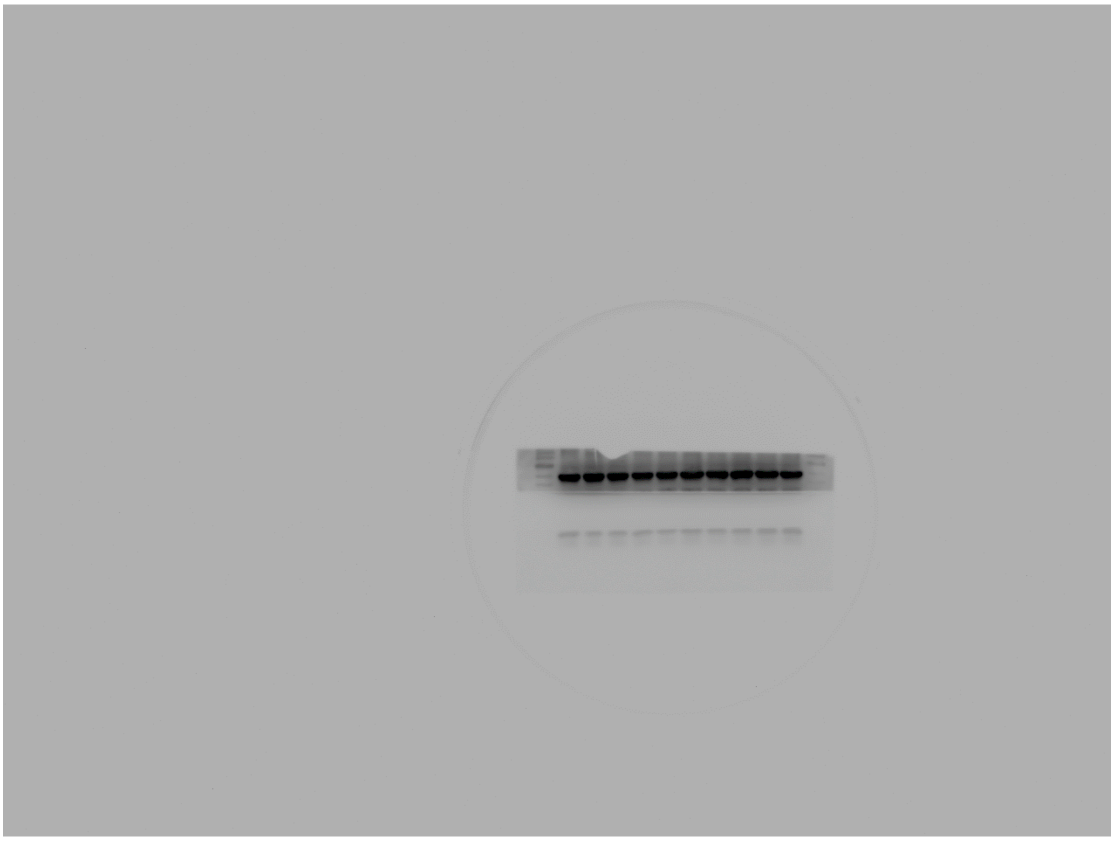

Fig3g gapdh (the up blot)

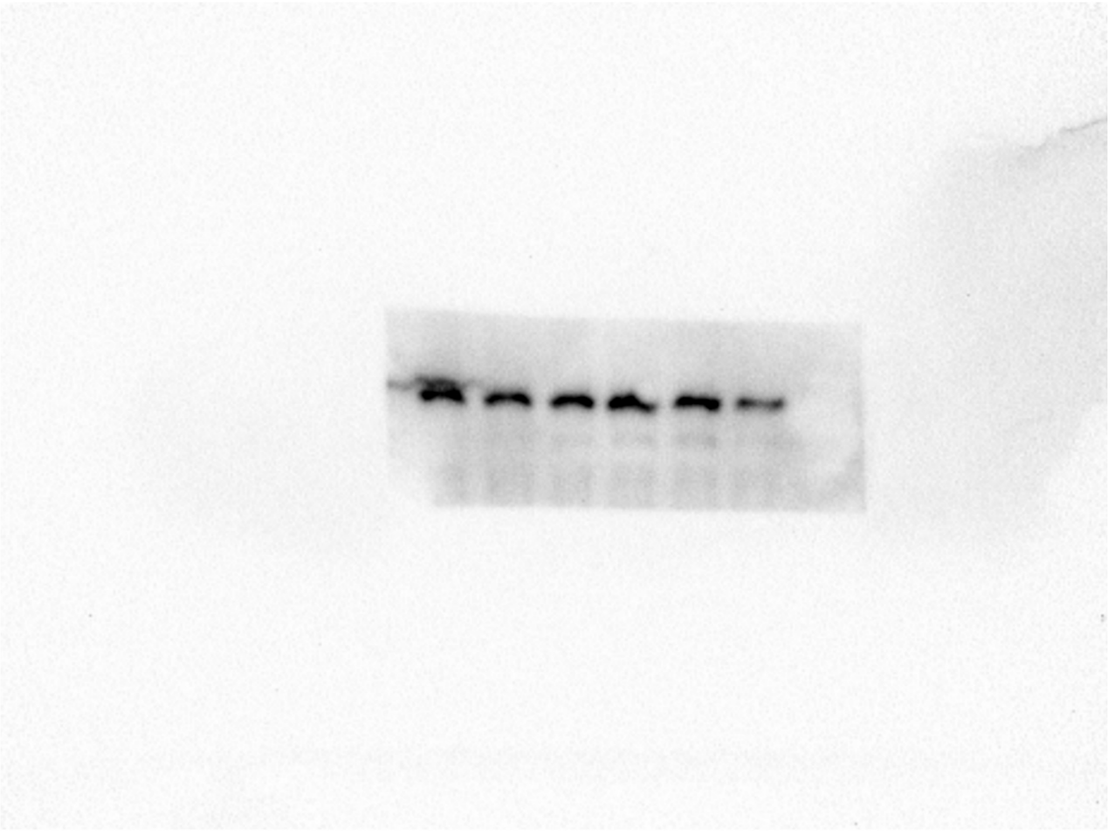

Fig4d egr3

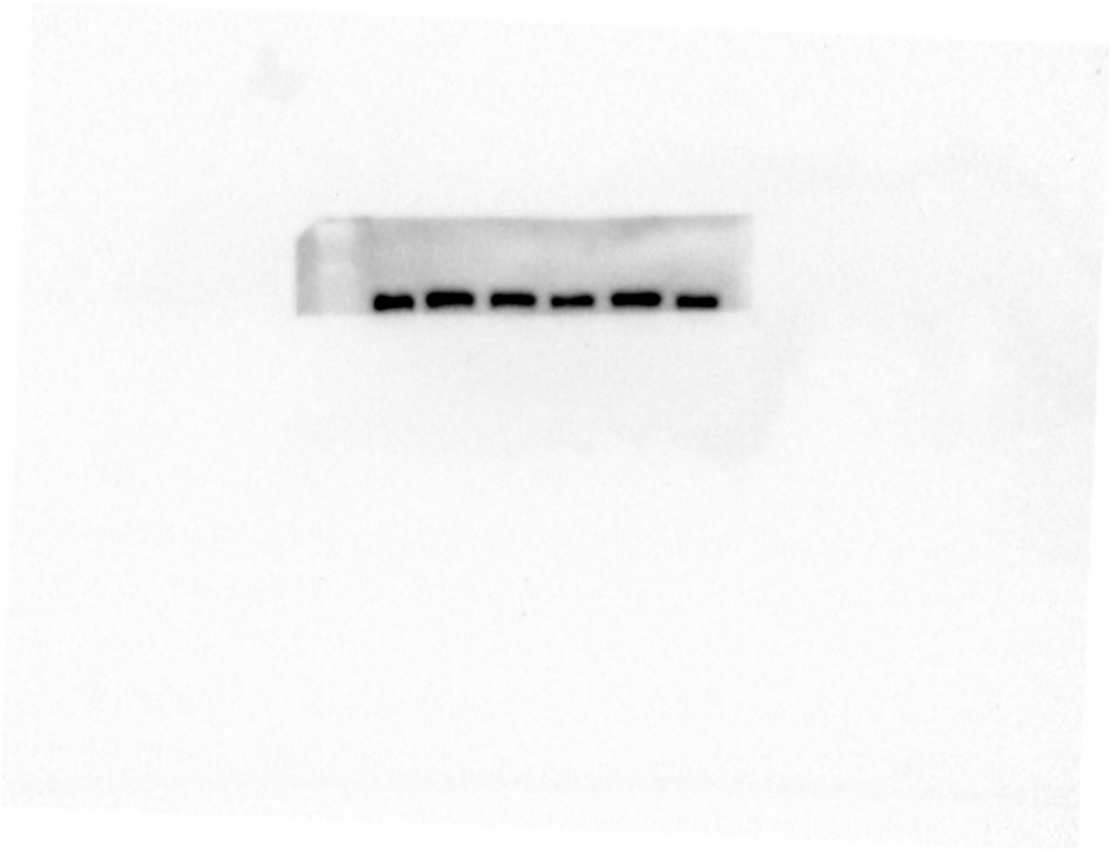

Fig4d gapdh

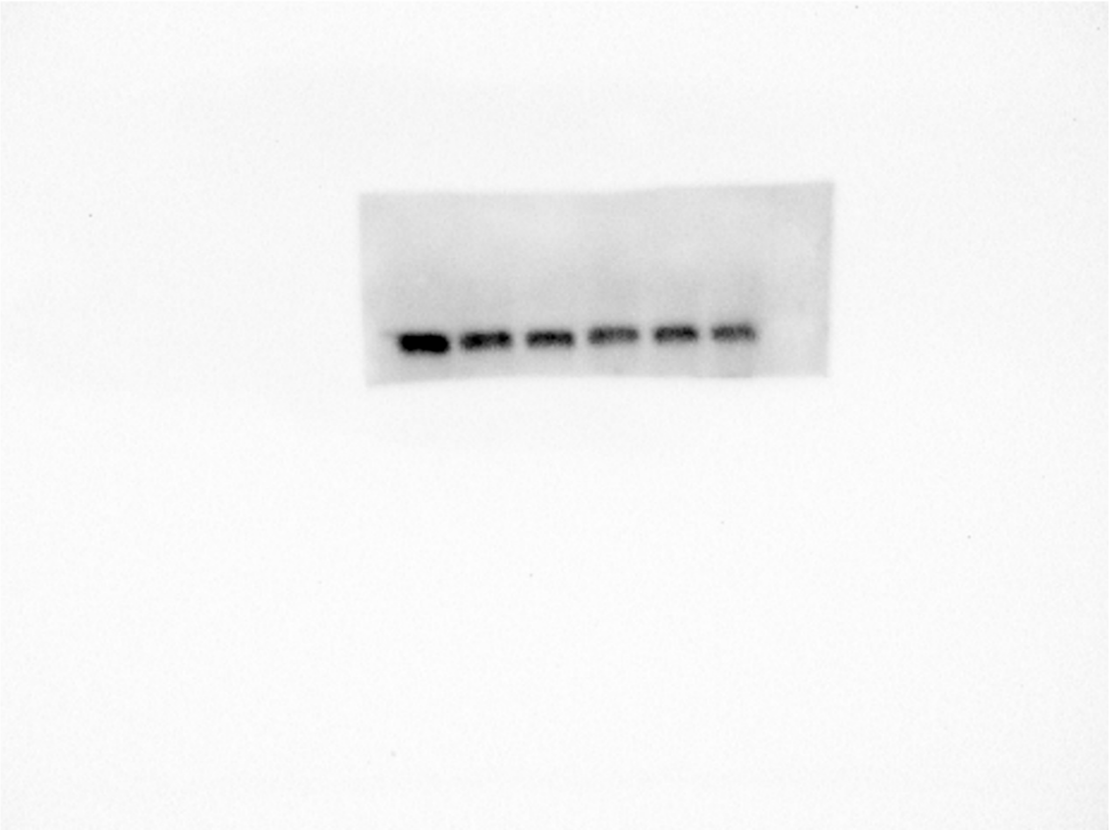

Fig4e egr3

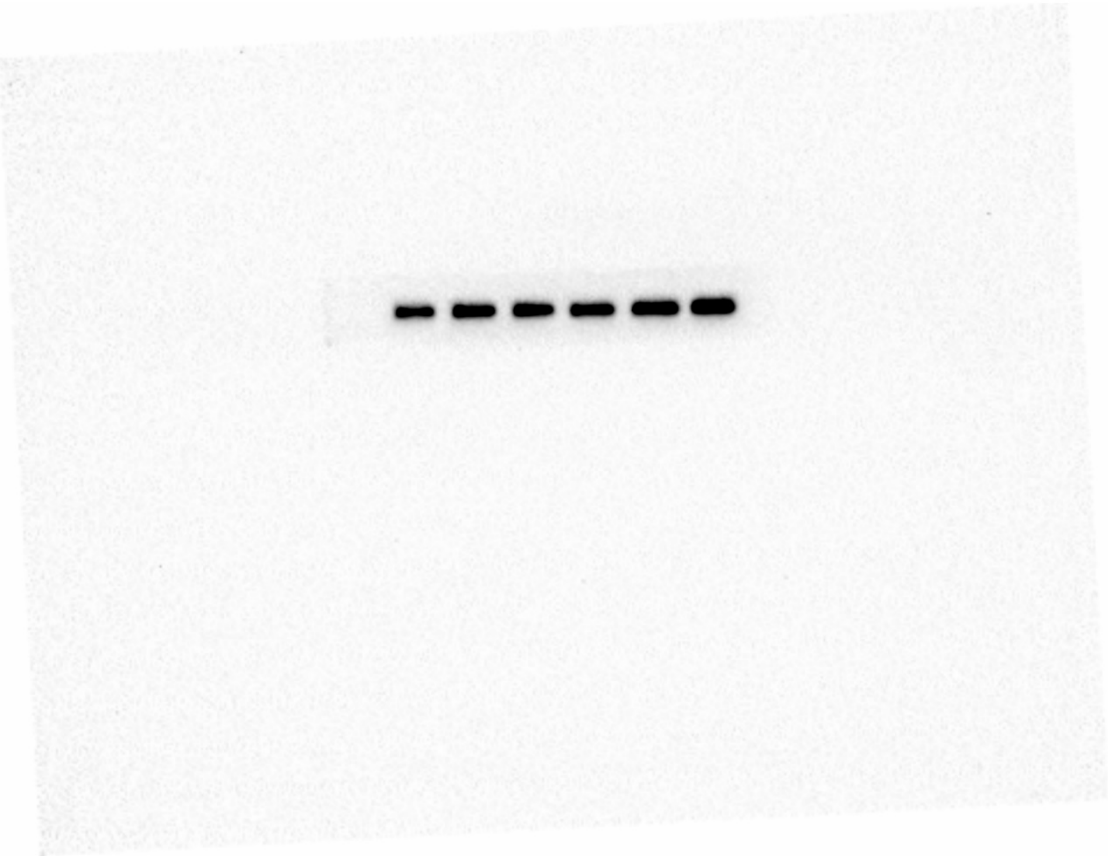

Fig4e gapdh

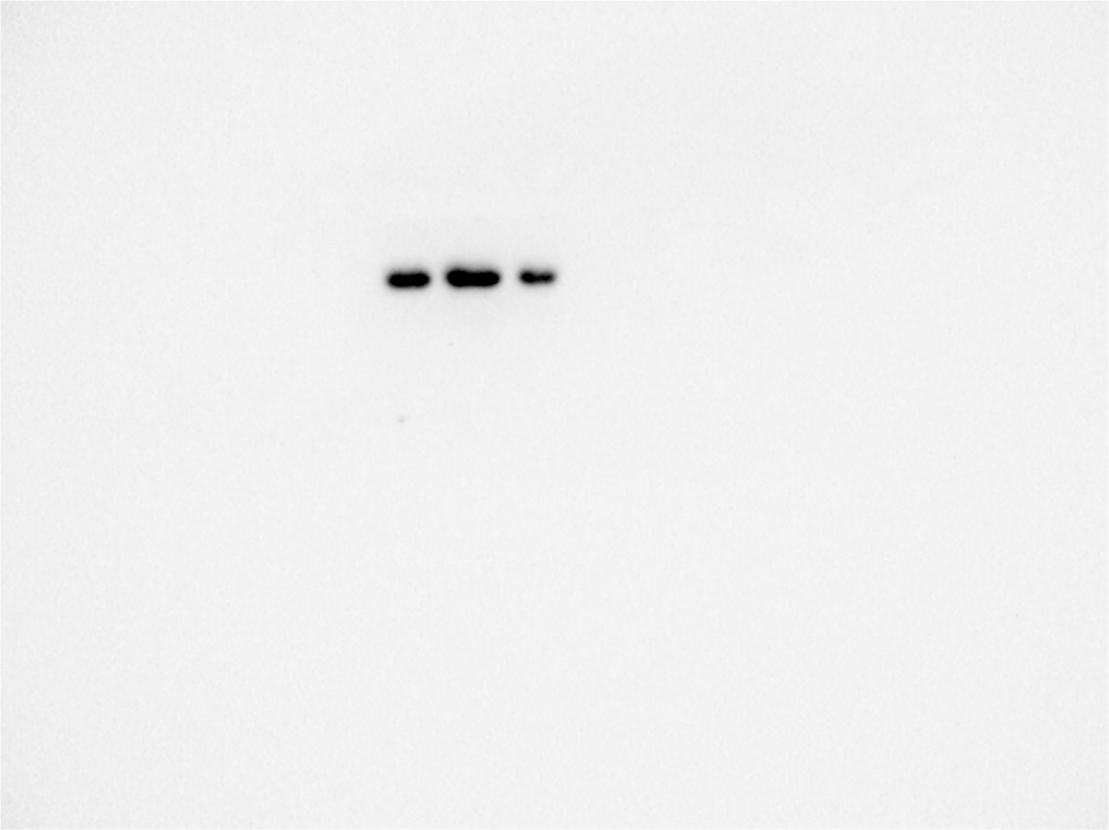

Fig 4h egr3

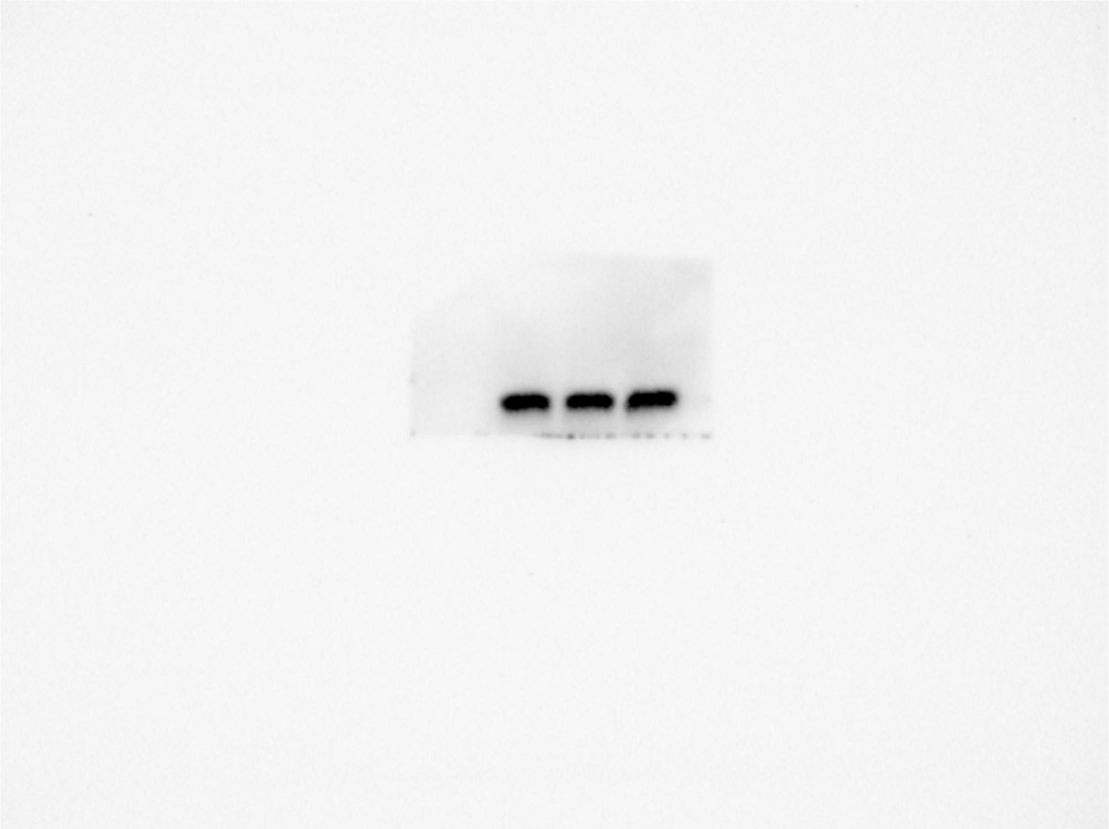

Fig 4h gapdh

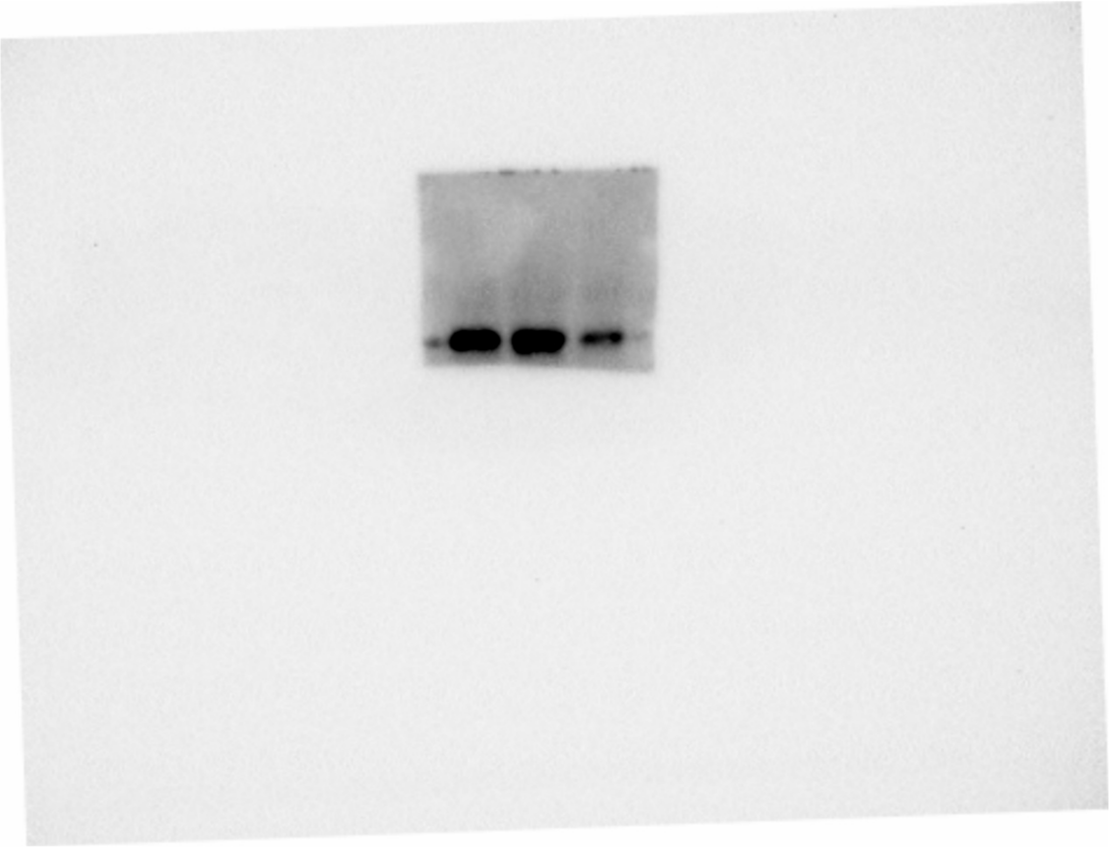

Fig4i egr3

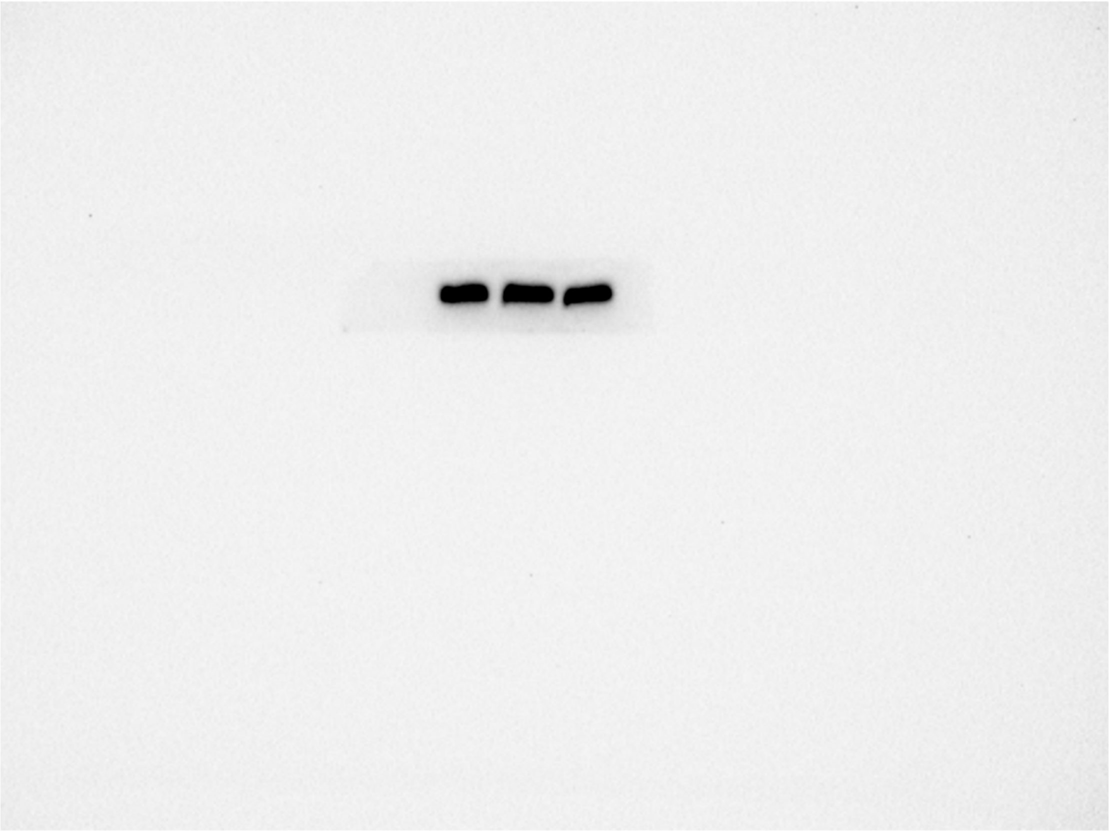

Fig4i gapdh

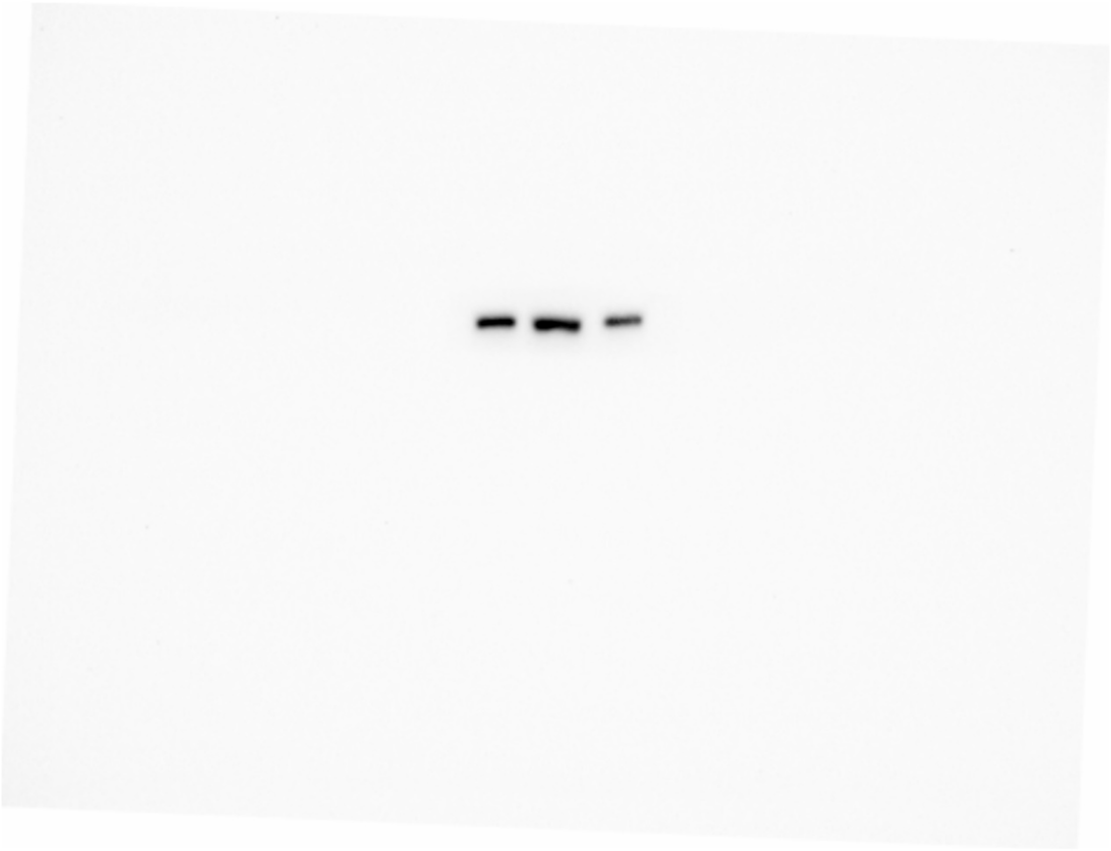

Fig 4l egr3

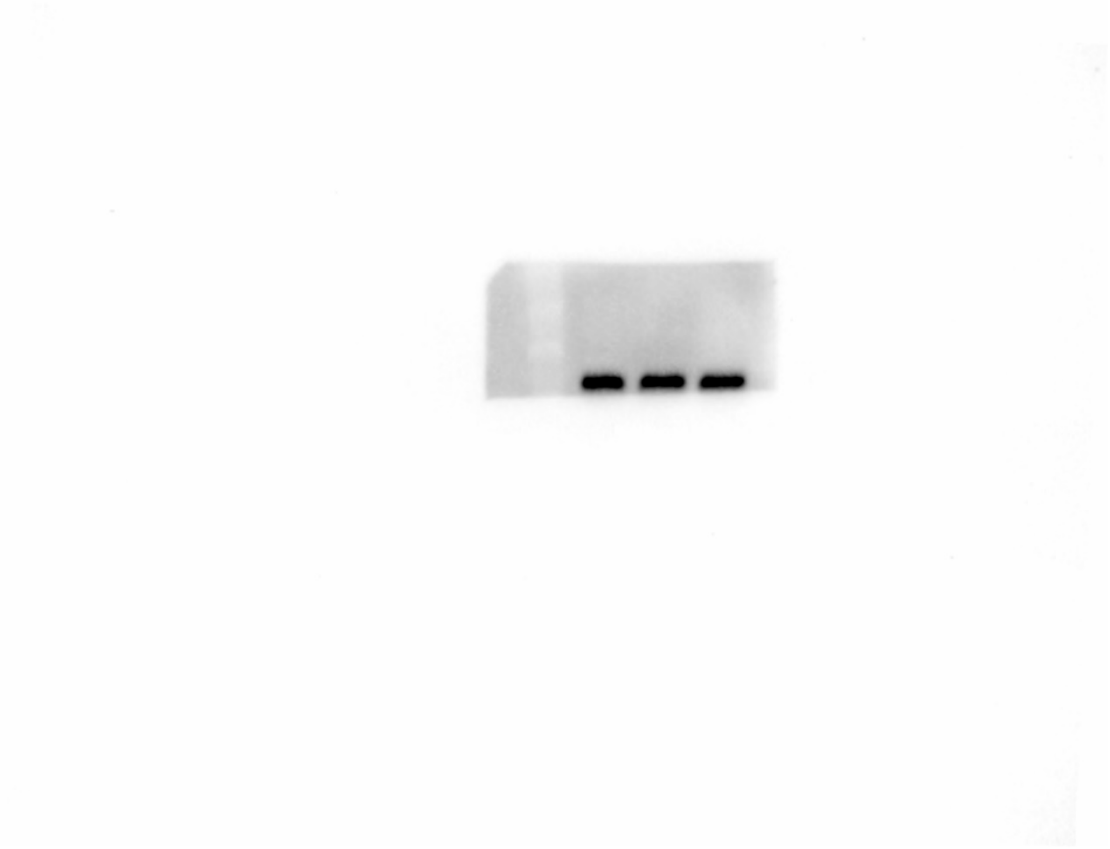

Fig4l gapdh

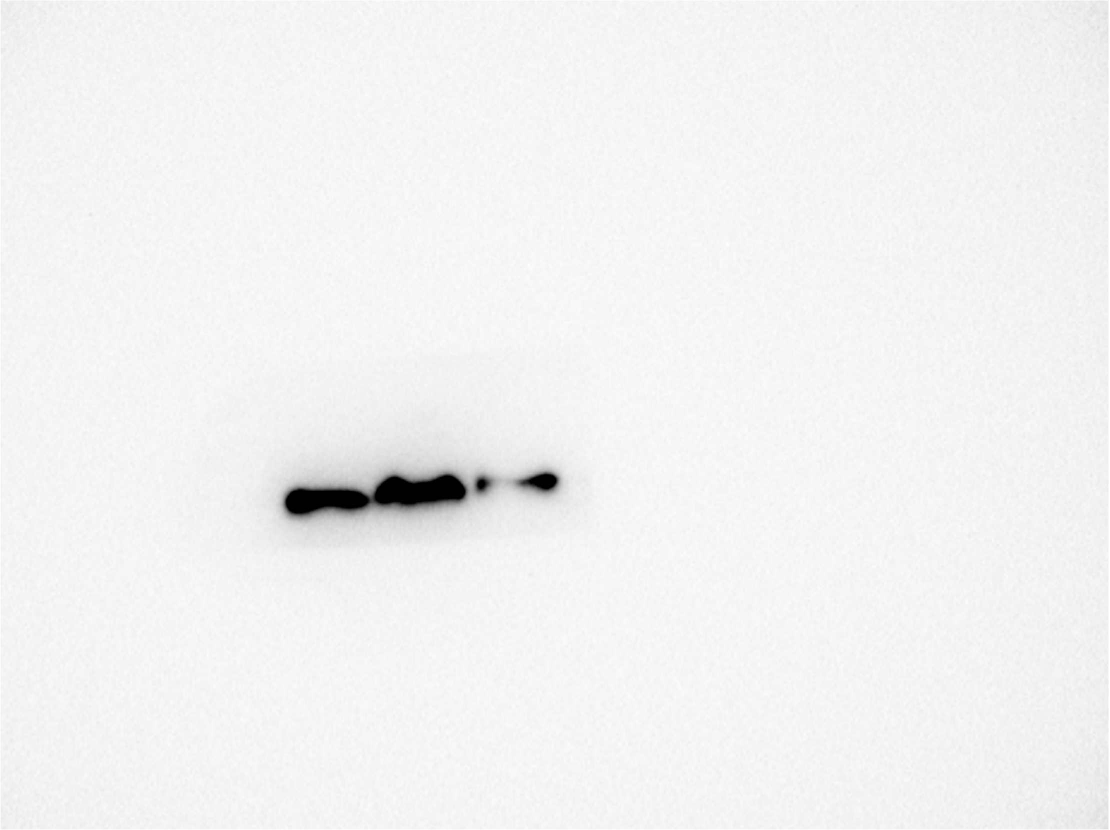

Fig4m egr3

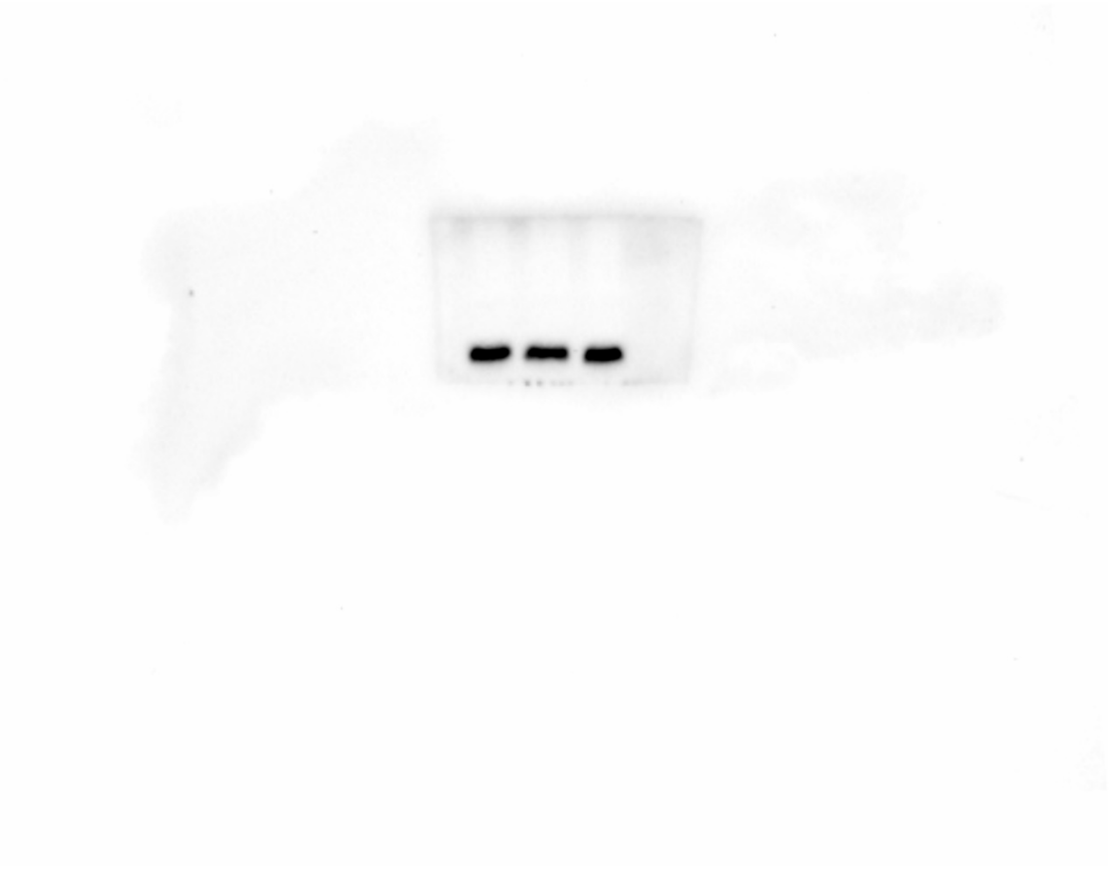

Fig4m gapdh

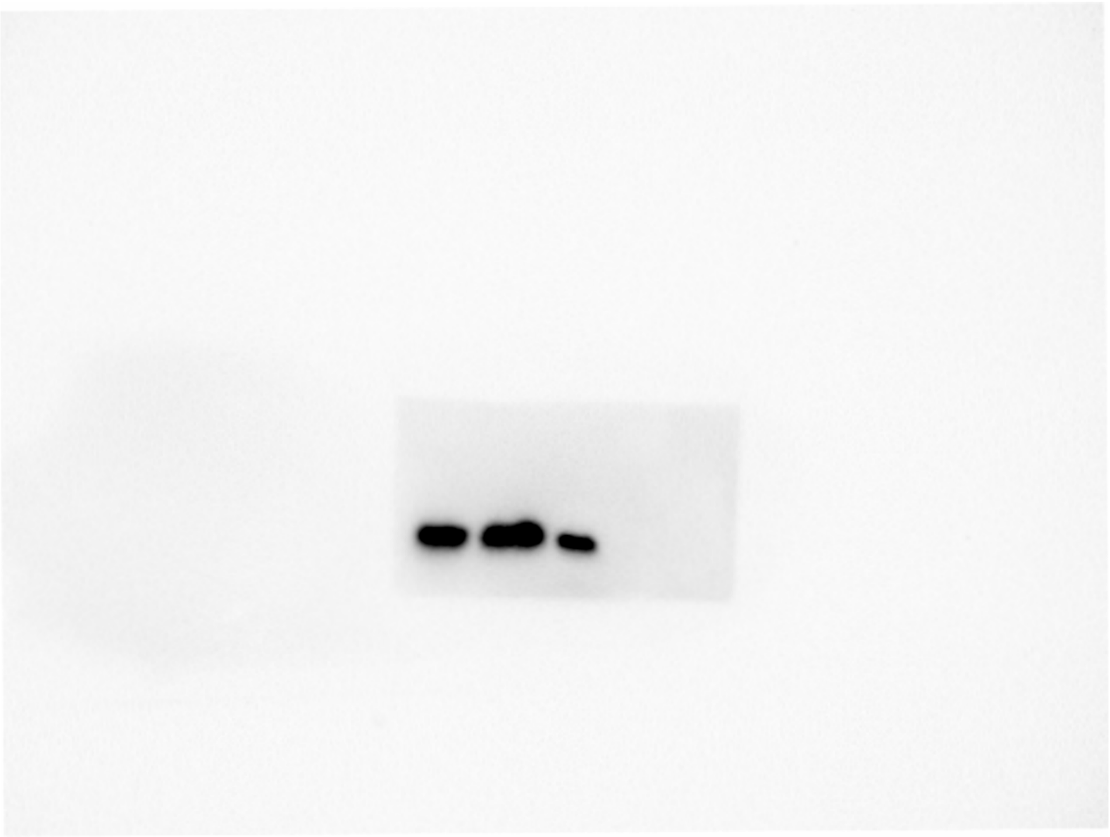

Fig6a cidec

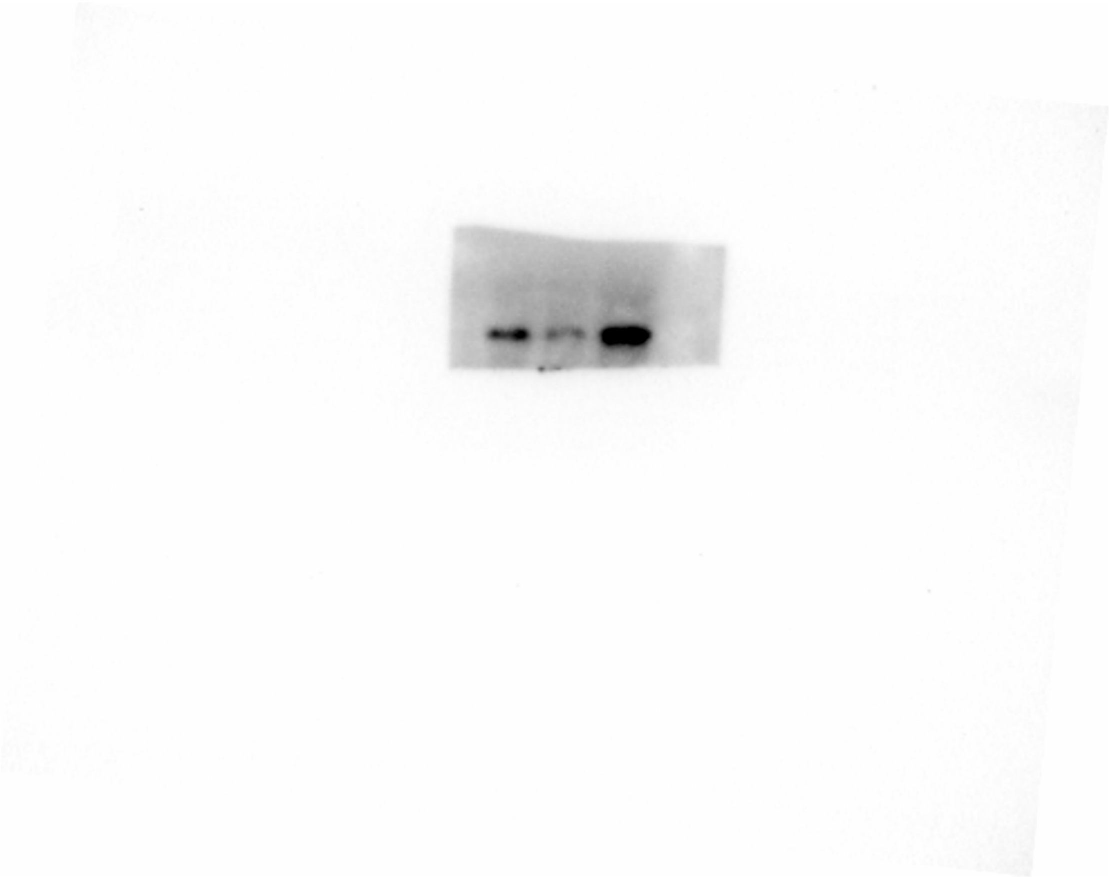

Fig 6a egr3

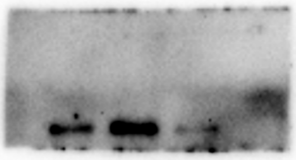

Fig6a fabp4

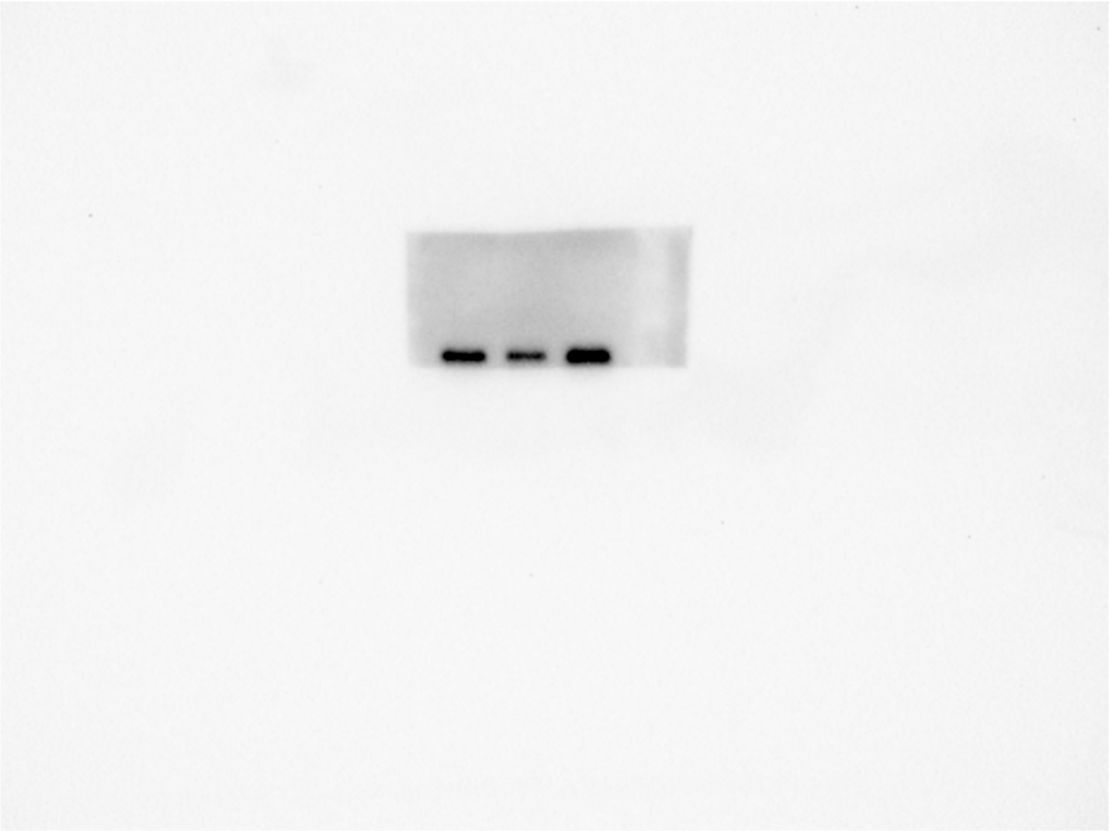

Fig6a hdac6

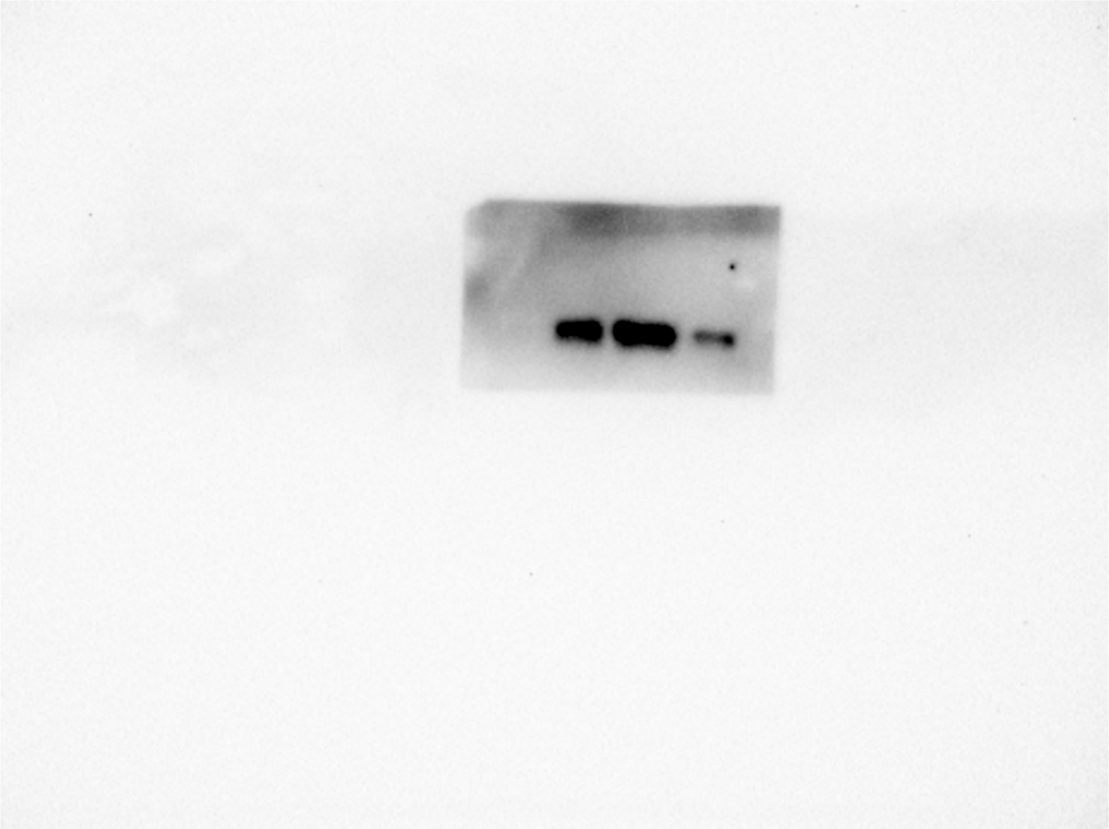

Fig6a ppar

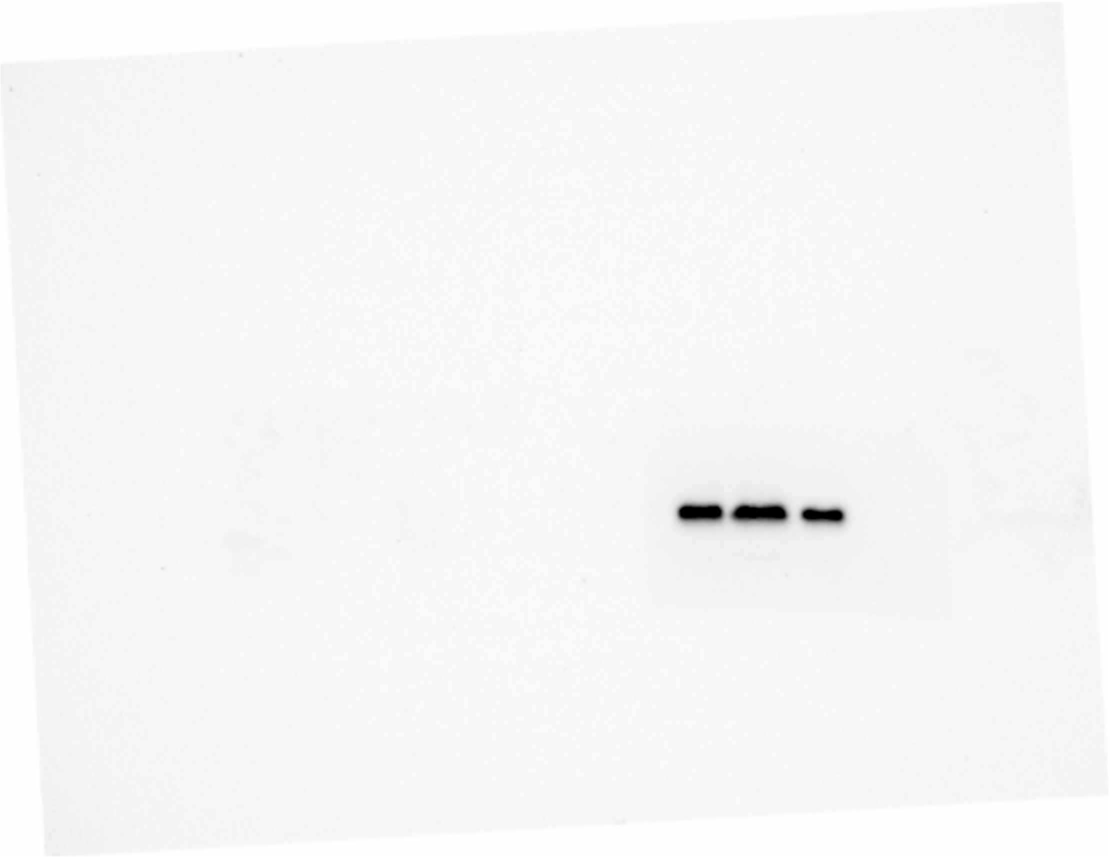

Fig6e cidec

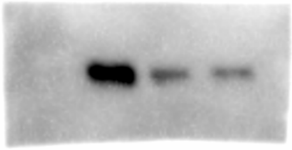

Fig6 e egr3

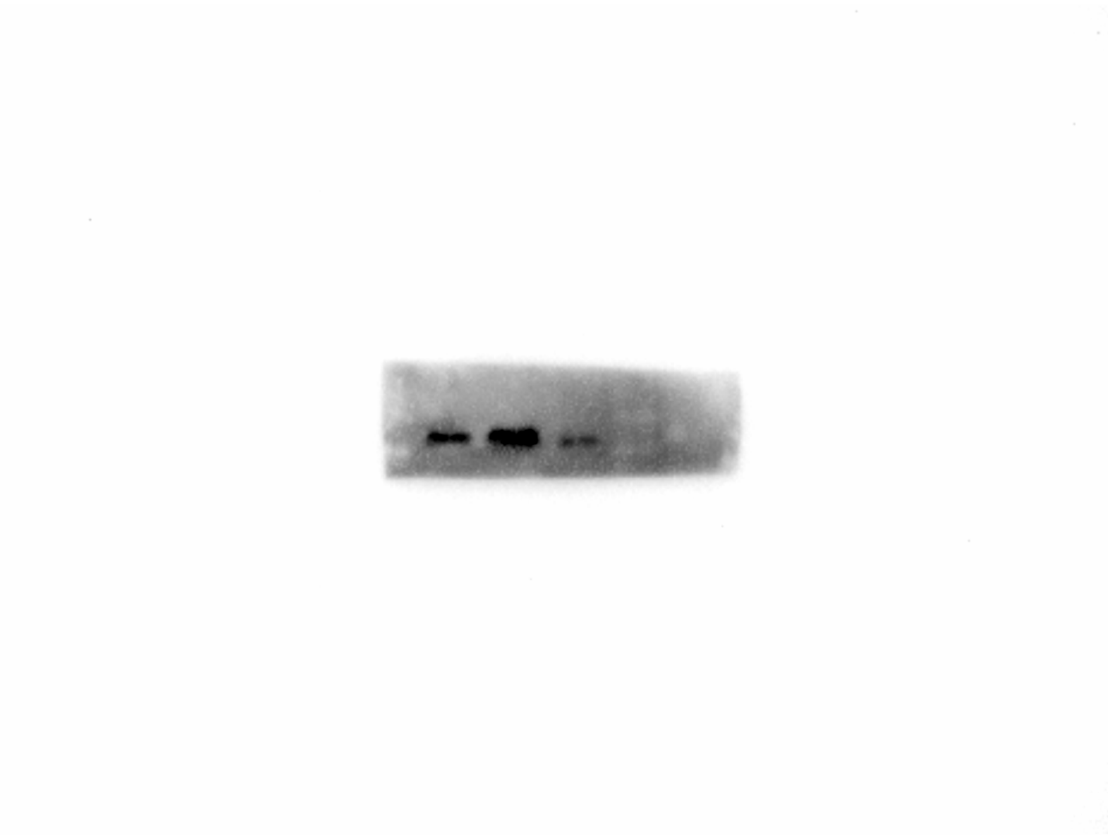

Fig 6e fabp4

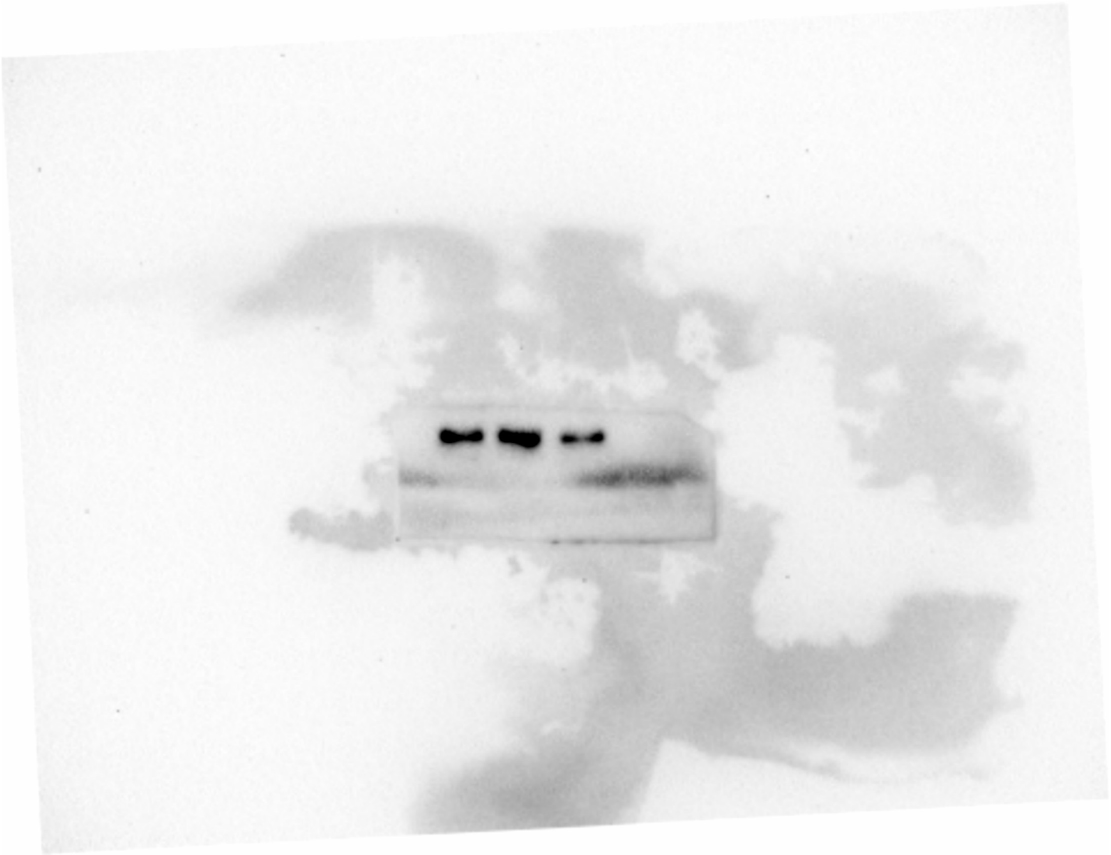

Fig6e ppar

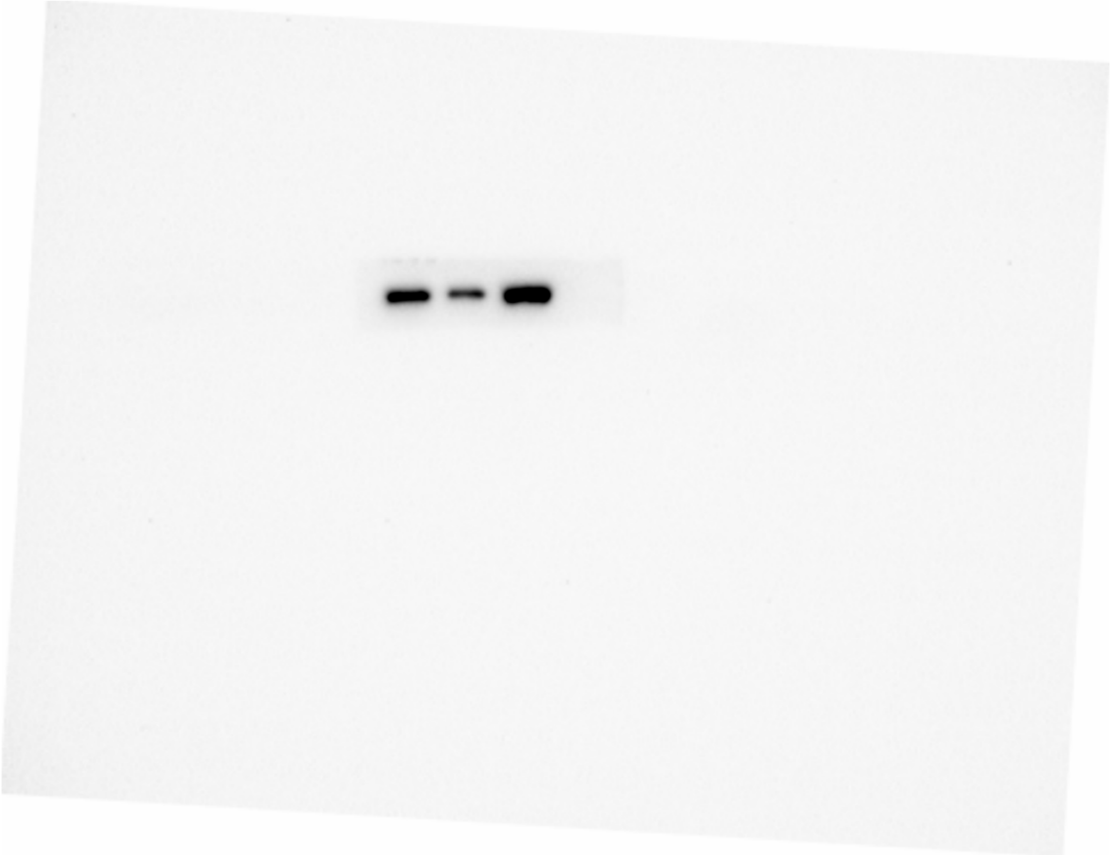

Fig6e hdac6

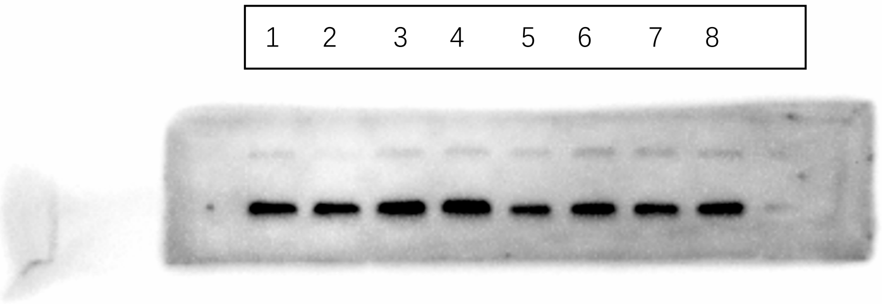

fig6a gapdh left 1-3, fig6e gapdh left 6-8
