# Supplementary material for: Shift work promotes adipogenesis via cortisol-dependent downregulation of EGR3-HDAC6 pathway
Source: Cell Death Discov. 2024 Mar 11;10:129. doi: 10.1038/s41420-024-01904-9 (PMC10928160; doi:10.1038/s41420-024-01904-9)
Supplement: Supplementary file 2 — supplementary table1 [file 41420_2024_1904_MOESM2_ESM.docx]

**Supplementary Table 1 Primer sequences used for qRT-PCR**

| **Gene name** | **Primer sequence (5’-3’)** |
| --- | --- |
| hEGR3 | 5’-GACATCGGTCTGACCAACGAG-3’ |
|  | 5’-GGCGAACTTTCCCAAGTAGGT-3’ |
| mEGR3 | 5’-TTGCCTGACAATCTGTACCCC-3’ |
|  | 5’-TAATGGGCTACCGAGTCGCT-3’ |
| hC/EBPα | 5’-TGGACAAGAACAGCAACGAG-3’ |
|  | 5’-TTGTCACTGGTCAGCTCCAG-3’ |
| mC/EBPα | 5’-CAAGAACAGCAACGAGTACCG-3’ |
|  | 5’-GTCACTGGTCAACTCCAGCAC-3’ |
| hPPARγ | 5’- GAGAAGACTCAGCTCTAC-3’ |
|  | 5’- CAAGCATGAACTCCATAGTG-3’ |
| mPPARγ | 5'-TCGCTGATGCACTGCCTATG-3’ |
|  | 5’-GAGAGGTCCACAGAGCTGATT-3’ |
| hFABP4 | 5’-AGCACCATAACCTTAGATGGGG-3’ |
|  | 5’- CGTGGAAGTGACGCCTTTCA-3’ |
| mFABP4 | 5’-AAGGTGAAGAGCATCATAACCCT-3’ |
|  | 5’-TCACGCCTTTCATAACACATTCC-3’ |
| mHDAC6 | 5’-TCCACCGGCCAAGATTCTTC-3’ |
|  | 5’-GCCTTTCTTCTTTACCTCCGCT-3’ |
| hGAPDH | 5’-AGCCAGGGTTGCACACTTT-3’ |
|  | 5’-CAGCCTTCTCCATGGTGGTGAAGA-3’ |
| mGAPDH | 5’- AGGTCGGTGTGAACGGATTTG-3’ |
|  | 5’-TGTAGACCATGTAGTTGAGGTCA-3’ |
